# Splicing-associated chromatin signatures: a combinatorial and position-dependent role for histone marks in splicing definition

**DOI:** 10.1038/s41467-021-20979-x

**Published:** 2021-01-29

**Authors:** E. Agirre, A. J. Oldfield, N. Bellora, A. Segelle, R. F. Luco

**Affiliations:** 1 Institute of Human Genetics, UMR9002 CNRS-University of Montpellier, 34000 Montpellier, France; 2 Institute of Nuclear Technologies for Health (INTECNUS), National Scientific and Technical Research Council (CONICET), Bariloche, 8400 Argentina; 3 Present Address: Laboratory of Molecular Neurobiology, Department of Medical Biochemistry and Biophysics, Karolinska Institutet, Stockholm, Sweden

**Keywords:** Machine learning, Histone post-translational modifications, Alternative splicing

## Abstract

Alternative splicing relies on the combinatorial recruitment of splicing regulators to specific RNA binding sites. Chromatin has been shown to impact this recruitment. However, a limited number of histone marks have been studied at a global level. In this work, a machine learning approach, applied to extensive epigenomics datasets in human H1 embryonic stem cells and IMR90 foetal fibroblasts, has identified eleven chromatin modifications that differentially mark alternatively spliced exons depending on the level of exon inclusion. These marks act in a combinatorial and position-dependent way, creating characteristic splicing-associated chromatin signatures (SACS). In support of a functional role for SACS in coordinating splicing regulation, changes in the alternative splicing of SACS-marked exons between ten different cell lines correlate with changes in SACS enrichment levels and recruitment of the splicing regulators predicted by RNA motif search analysis. We propose the dynamic nature of chromatin modifications as a mechanism to rapidly fine-tune alternative splicing when necessary.

## Abstract (web summary)

Chromatin is known to regulate splicing by modulating recruitment of splicing factors. Using machine learning approaches, the authors have underlined a chromatin code for alternative splicing regulation that is conserved amongst cell lines.

## Introduction

An essential aspect of cell identity is to express the right subset of proteins, at the right developmental stage, and to maintain this expression pattern as a cell memory. It is not only about which gene is expressed, but also how it is processed by mechanisms such as alternative splicing. Alternative splicing is a highly regulated process that affects most human genes. It consists in the alternative processing of a molecule of pre-mRNA into different mature mRNAs, and therefore coding proteins, thereby increasing genome diversity and complexity^1,2^. Alternatively spliced exons are defined by cis-regulatory sequences, called RNA motifs, responsible for the recruitment of positive and negative trans-acting factors that will favour or inhibit the inclusion of the regulated exon in the pre-mRNA^3,4^. The strength and composition of these RNA binding sites, the differential G/C content between introns and exons, RNA secondary structures and exon/intron lengths play an important role in predicting exon inclusion^5–8^. On top of that, splicing is mostly a co-transcriptional process in which chromatin conformation, histone modifications, DNA methylation and transcriptional regulators have also been shown to impact the final splicing outcome^9–20^. Effectively, nucleosomes have been shown in genome-wide studies to non-randomly distribute along genes, with a specific positioning at the intron/exon junction, creating transcriptional roadblocks that can shape the final splicing outcome^16,21–25^. Moreover, these exon-specific nucleosomes are enriched in characteristic histone modifications, many of which have already been shown by our group and others to play an active role in the final splicing decision^22–30^. So far, there are two models functionally linking chromatin to splicing. The kinetic model, in which by slowing down the RNA polymerase II, chromatin modulates the window of time for splicing regulators to bind to competing RNA binding sites; and the recruitment model, in which chromatin modifications modulate splicing factors binding to the pre-mRNA via recruitment of chromatin binding proteins that act as adaptors between the chromatin and the splicing machinery^9^.

There have been several attempts to identify all the chromatin modifications that are differentially enriched along alternatively spliced exons^11,19,31–33^. Recently, several works have started to functionally link histone modifications with splicing at a genome-wide level. First, chronic cocaine administration in mice was shown to induce dramatic changes in chromatin and the alternative splicing of genes from the nucleus accumbens brain reward region through physical interaction of the splicing factor A2BP1 (RBFOX1) with H3K4me3 at target genes^34^. In a disease context, downregulation of the H3K79 methyltransferase DOT1L1 in two acute myeloid leukaemia cell lines induced inclusion of H3K79me2-marked exons, which reduced cell proliferation and transformation^35^. Finally, in a developmental context, half of the alternatively spliced events that change upon human embryonic stem cell differentiation were shown to be differentially enriched in H3K36me3, H3K27ac or H4K8ac^36^. However, in most of these studies, just the histone marks of interest were analysed individually, omitting many known chromatin marks and their combinatorial role in gene expression. Moreover, splicing has been studied in a binomial way, with exons either totally included or excluded from the mRNA, without taking in consideration the real complexity of alternative splicing, in which different spliced mRNA isoforms co-exist in the same cell at different levels.

In this study, we have used a supervised machine learning approach to identify all the chromatin modifications that could classify alternatively spliced exons into four splicing groups based on exon-inclusion levels, from highly excluded (0% exon inclusion) to highly included (100% exon inclusion). From the 26 chromatin modifications analysed, 11 were shown to differentially mark 34% of all the alternatively spliced exons analysed in H1 human embryonic cells. When studied individually, there was no obvious association between enrichment of a specific histone mark and a percentage of exon inclusion. However, when studied in a combinatorial way, we found seven unique combinations of chromatin marks, co-enriched at a specific position along the regulated exon. These chromatin marks were selectively marking exons with a specific range of exon inclusion levels, creating like this what we called splicing-associated chromatin signatures (SACS). Interestingly, alternatively spliced exons marked by these SACS were smaller than constitutive, had distinctive gene ontology functions and characteristic RNA binding motifs, suggesting that each chromatin signature might coordinate the recruitment of specific splicing regulators to the pre-mRNA of a subset of genes that share common functional pathways. As expected, a shift in exon inclusion levels between two different cell lines correlated with a change in histone marks enrichment levels and binding of the corresponding splicing regulator, as predicted by the SACS model, further supporting a functional link between chromatin and cell-specific alternative splicing.

## Results

### Exon-specific chromatin modifications can discriminate between different levels of exon inclusion

**Fig. 1:**
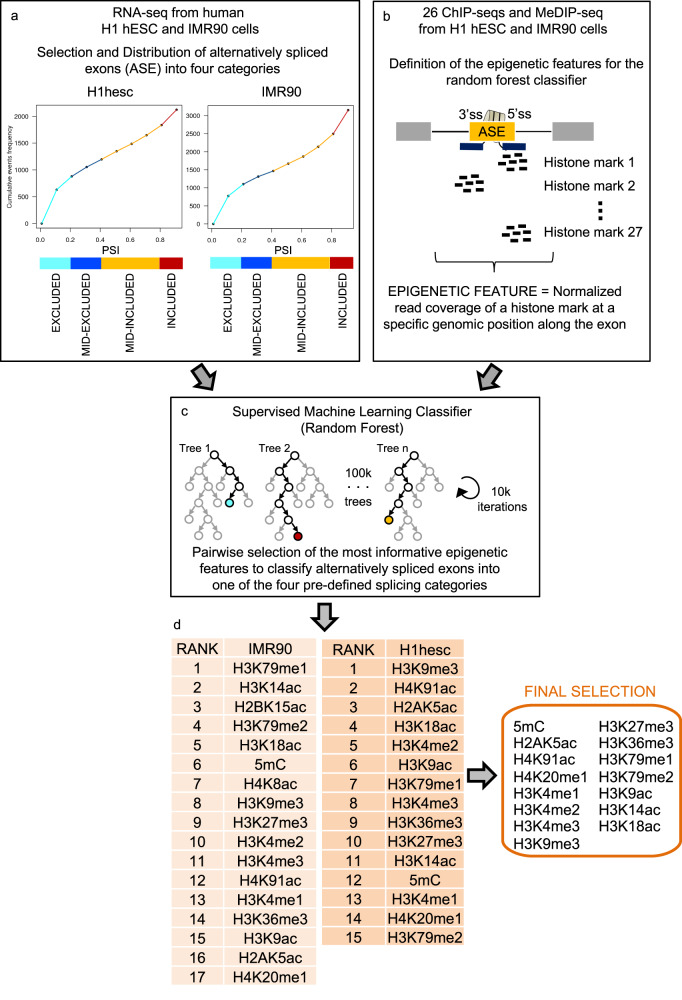
Schematic pipeline of the machine learning approach used to identify the chromatin modifications that can classify exons into four different splicing categories. **a** Cumulative distribution of alternatively spliced exons in human H1 embryonic stem cells and IMR90 foetal fibroblasts using available RNA-seq datasets. Four arbitrary groups were created based on the percentage of exon inclusion (PSI). A colour code was given to each category, with light blue for well excluded (0 < PSI < 0.2), dark blue for mid-excluded (0.2 < PSI < 0.4), orange for mid-included (0.4 < PSI < 0.8) and red for well included (0.8 < PSI < 1) events. **b** The enrichment levels of 26 histone marks and DNA methylation levels around the 3′ and 5′ splice sites (ss) of alternatively spliced exons were calculated and defined as epigenetic features using available ChIP-seq and MeDIP-seq data from the ENCODE and Roadmap Epigenomics Projects. **c** A Random Forest classifier (with 100,000 trees and 10,000 iterations) was applied to all the binary comparisons between the four splicing categories to identify the epigenetic features that were most informative to classify the selected splicing events into the four pre-defined splicing categories in H1 and IMR90 cells. **d** The epigenetic features informative to classify splicing events into any of the four pre-defined splicing groups in H1 and IMR90 cells were ranked by importance. A final list of chromatin modifications found in common between the two cell lines is shown in the right. The same analyses using randomised splicing levels did not select any feature. More details of the random forest results are summarised in Supplementary Data 2.

Splicing is mainly a co-transcriptional process in which chromatin and transcriptional regulators have long been shown to impact the final splicing outcome at a number of model genes^9,14,20,37^. However, a systematic approach to identify all the histone modifications that can influence alternative splicing at a genome-wide level is lacking. Neither do we know whether these histone marks can act in a combinatorial way and what these chromatin-marked spliced genes have in common. To address those questions, we took advantage of publicly available transcriptomics and epigenomics data from the Roadmap Epigenomics and ENCODE projects to identify, by using machine learning approaches, the chromatin modifications that were informative to classify alternatively spliced exons in different splicing groups (Fig. 1 and Supplementary Data 1). We first selected in two cell lines in which the most extensive epigenomics data is available, which are human H1 embryonic stem cells and IMR90 foetal lung fibroblasts, splicing events in which an alternatively spliced exon was flanked by two constitutive exons. Then we distributed these cassette exons into different splicing groups based on the percentage of exon inclusion, using the Percent Spliced In index (PSI). Most of the studies done until now study splicing as a binomial process, in which exons are either totally included (PSI > 80%) or excluded (PSI < 20%). However, a mix population of splicing isoforms, with the exon included in some transcripts and excluded in some others, can also co-exist in the same cell or population of cells, increasing the splicing complexity. Assuming that the mechanisms of splicing regulation might be different between these splicing conditions, we decided to distribute the alternatively spliced exons into four categories, with highly included (PSI > 80%), mid-included (40% < PSI < 80%), mid-excluded (20% < PSI < 40%) and highly excluded events (PSI < 20%) (Fig. 1a). As a control, we used a random selection of constitutively spliced exons, coming from the same genes as the selected alternatively spliced exons, to avoid genomic differences between the groups. An exon was considered constitutive when it is included at a PSI > 95% in more than 75% of the ten cell lines analysed and is annotated as constitutive in Ensembl72 (BioMart). Lowly expressed genes (Transcripts per million reads (TPM) < 10) and the two first exons from each alternatively spliced gene were excluded from the analysis to avoid a chromatin effect from the transcription start sites. Then, using available chromatin immunoprecipitation (ChIP-seq) and methyl DNA immunoprecipitation high-throughput sequencing data (MeDIP-seq), we calculated the levels of 26 histone modifications and methyl DNA (5mC) at two regions around the start (3’ss) and the end (5’ss) of each alternatively spliced exon which were used as epigenetic features for downstream analysis (Fig. 1b). Finally, using a supervised Random Forest-based classifier^38^, we identified the epigenetic features with higher importance to classify exons into the four pre-defined splicing groups in H1hesc and IMR90 cells, independently (Fig. 1c, Supplementary Fig. 1 and Supplementary Data 2). From the ranked features, fourteen histone modifications and DNA methylation were found in common between the two cell lines (Fig. 1d). Whereas not a single chromatin modification was capable of classifying alternatively spliced exons randomly distributed into different splicing groups (Supplementary Fig. 1g). Half of these histone marks, H3K36me3, H4K4me3, H3K9me3, H3K9ac, H3K27me3 and DNA methylation, are well-known to play a role in alternative splicing^12,13,18,25–27,29,30,36^. However more novel histone marks were also found, like H2AK5ac, H4K91ac, H3K18ac, H3K14ac, H3K4me1, H3K20me1 and H3K79me1,2, which broadens the potential role of chromatin modifications in alternative splicing. Since histone marks are known to play a combinatorial role in gene expression, we next tested whether splicing-associated chromatin marks were co-enriched at alternatively spliced exons.

### Splicing-associated chromatin marks act in a combinatorial and position-dependent manner along the regulated exon

**Fig. 2:**
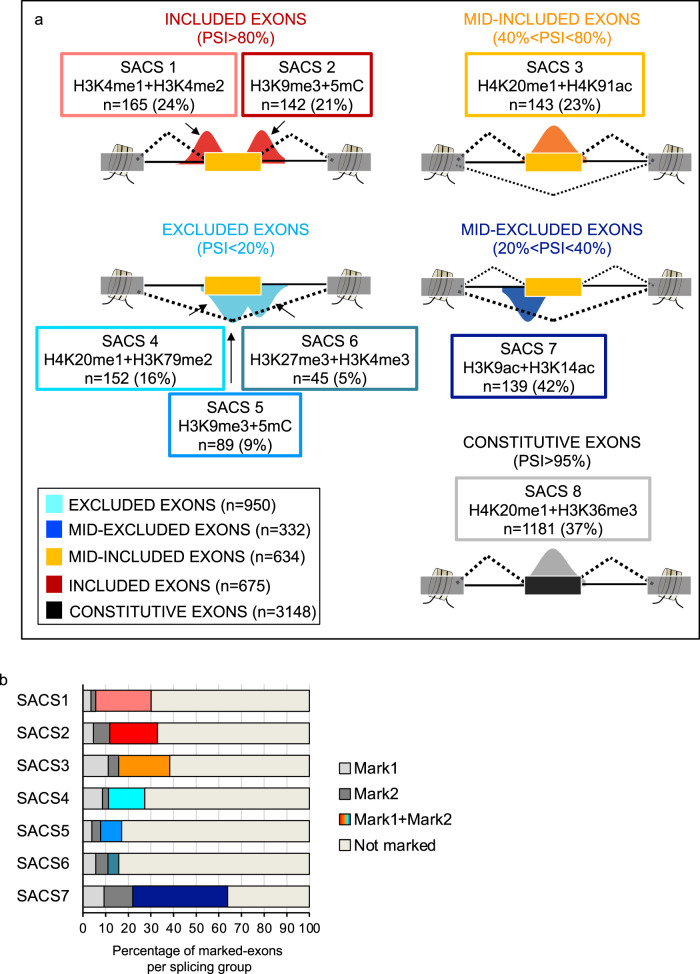
Splicing-associated chromatin signatures (SACS) in H1 hESCs. **a** Schematic representation of the seven combinations of chromatin modifications (SACS) that differentially mark alternatively spliced exons. As controls, we used exons with randomised splicing levels and constitutive exons, which are exons always included in the mRNA, from the same genes as the alternatively spliced exons analysed. For each SACS, we specify the splicing group it is related to, the two co-enriched histone marks, the position of enrichment along the exon (represented by a peak) and the total number (*n*) of exons marked by the chromatin signature (in brackets the percentage of chromatin-marked exons respect the total number of exons analysed per group). **b** Percentage of alternatively spliced exons marked by the two chromatin modifications defining a SACS, just one of the two marks or none.

When comparing histone modification levels at the selected cassette exons, we could not find many striking differences between the four pre-defined splicing groups (Supplementary Fig. 2). Mid-excluded exons are slightly more enriched in 5mC and histone acetyl marks, such as H2AK5ac, H3K14ac, H3K18ac, H4K91ac, as predicted by the Random Forest (Supplementary Figs. 1b, e, f and 2). Whereas included exons have reduced levels in H3K27me3, H3K36me3, H3K79me2, H3K9me3 (Supplementary Figs. 1a, b, c and 2). However, when studying the co-occurrence by pairs of these same chromatin modifications at specific positions around the selected exons (Supplementary Data 3), we found that alternatively spliced exons are significantly marked by seven unique combinations of chromatin modifications that are specific to a splicing group and position along the regulated exon, creating like this what we called splicing-associated chromatin signatures (SACS) (Fig. 2a, Supplementary Fig. 3a–f and Supplementary Data 3). These chromatin signatures were selected based on two criteria: (1) there is a reciprocal and localised enrichment of two different chromatin marks at a specific position around the exon, which can be either upstream, at the body or downstream the regulated exon. We started looking for co-enrichment of just two marks for simplicity, more complex combinatorial patterns might certainly exist. (2) the localised chromatin signature is specific for a splicing category and is not significantly found at any of the other splicing groups analysed, including constitutive exons nor exons with randomised inclusion levels (Fig. 2a, Supplementary Fig. 3a–f and Supplementary Data 3). In H1 hESCs, almost 900 alternatively spliced exons (34% from all the exons analysed) are marked by one of these chromatin signatures (SACS) (Fig. 2a and Supplementary Data 4). Importantly, when counting the number of alternatively spliced exons in H1 cells marked by a SACS or just one of the two epigenetic modifications defining the SACS, except for SACS6, the combinatorial effect was always dominant over the presence of just an individual mark, supporting an additive value of SACS to discriminate between splicing groups (Fig. 2b). Surprisingly, chromatin modifications do not only differentially mark exons homogenously included (PSI > 80%) or excluded (PSI < 20%) in a single splicing isoform, but they also mark exons present in more than one splicing isoform, leading to a mixed percentage of exon inclusion (20% < PSI < 80%). Effectively, almost half of all the mid-excluded exons, in which the most abundant variant is the excluded one (20% < PSI < 40%), are enriched in H3K14ac and H3K9ac upstream the exon (SACS7); whereas a fourth of the mid-included exons (40% < PSI < 80%), in which the most abundant isoform is the included one, are enriched in H4K20me1 and H4K91ac at the exon body (SACS3; Fig. 2a and Supplementary Fig. 3c, f). This differential marking suggests that chromatin might not only influence alternative splicing, but also isoform stoichiometry, which could impact cell’s proteome diversity and capacity to respond to a specific stimulus.

Interestingly, when looking in more detail into these different splicing-associated chromatin signatures, we found that some groups with opposite splicing outcome, such as SACS3 and SACS4, share the same histone mark, H4K20me1. While others, such as SACS2 and SACS5, are differentially marked by the same modifications, but at different positions along the alternatively spliced region (Fig. 2a and Supplementary Fig. 3b–d). These results highlight a much more complex relationship between chromatin marks and splicing patterns that has been largely overlooked in most of the genome-wide studies done until now. In support of a functional role for the identified splicing-associated chromatin signatures, H3K9me3 + 5mC have also been shown to differentially co-regulate splicing in a context dependent way via recruitment of HP1 in embryonic stem cells^29^. Overexpression of H3K4me3 and H3K27me3 methyltransferases in human mesenchymal stem cells reduces inclusion of exon IIIc in *Fgfr2*^26,27^. The H4K91 acetyltransferase Gcn5 has been shown to favour exon inclusion by regulating the co-transcriptional recruitment of the core spliceosome protein U2 snRNP to the pre-mRNA^39^. Recently, H3K79me2 has been shown to induce exon exclusion in a subset of genes important for cell proliferation in two acute myeloid leukaemia cell lines^35^.

**Fig. 3:**
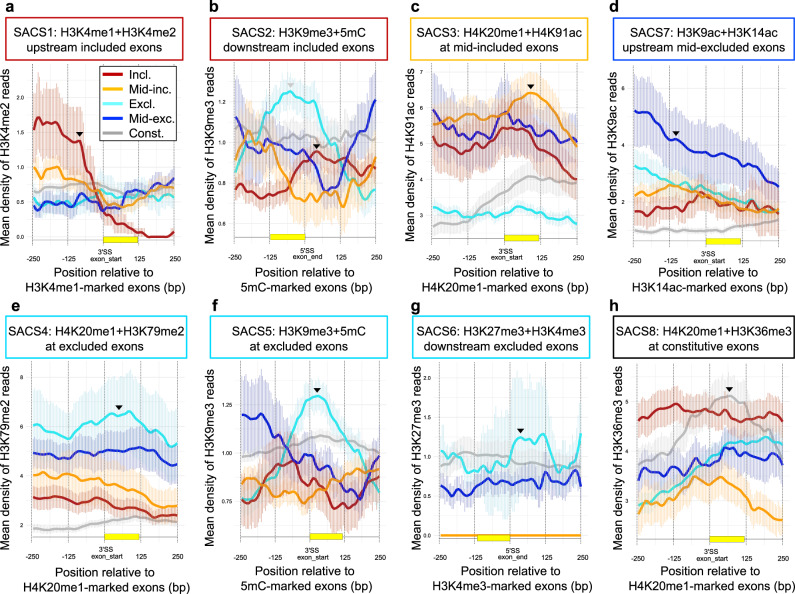
Profiles of Splicing-associated Chromatin Signature (SACS). **a** Density profiles of H3K4me2 reads around exons marked by H3K4me1 upstream the 3’ss exon start. **b** H3K9me3 reads around exons marked by 5mC downstream the 5’ss end of the exon. **c** H4K91ac reads around H4K20me1-marked exons. **d** H3K9ac reads around exons marked by H3K14ac upstream the exon start. **e** H3K79me2 reads around H4K20me1-marked exons. **f** H3K9me3 reads around 5mC-marked exons. **g** H3K27me3 reads around exons marked by H3K4me3 downstream the end of the exon. **h** H3K36me3 reads around H4K20me1-marked exons in excluded (excl., light blue), mid-excluded (mid-exc., dark blue), mid-included (mid-inc., yellow), included (incl., red) and constitutive (const., grey) exons using available ChIP-seq datasets from H1 hESCs. The average read count and ±SEM of histone marks’ reads is represented ±250 bp from either the 5′ or 3′ splice site (ss), depending on the SACS. For each mark, we highlight with a black arrow the splicing group that is the highest enriched at a specific position around the regulated exon, as defined by the SACS. Please notice that H3K9me3 + 5mC is enriched at both included (SACS2) and excluded (SACS5) exons but at different positions, this is why there are two arrows (in grey the enrichment that corresponds to the other SACS).

To visualise the splicing-dependent co-enrichment of the identified chromatin signatures, we assessed the levels of a specific histone mark along exons enriched in the other mark in all four splicing groups. As expected, we found that H3K4me2 is co-enriched upstream H3K4me1-marked exons when included (SACS1, Fig. 3a). H3K79me2 only peaks at H4K20me1-marked exons when excluded (SACS4, Fig. 3e), whereas H4K91ac is more enriched at H4K20me1-marked exons when mid-included (SACS3, Fig. 3c). Similarly, H3K9me3 is mostly enriched at 5mC-marked exons when excluded (SACS5, Fig. 3f), and shifts towards the end of the exon when included (SACS2, Fig. 3b). Finally, H3K9ac peaks upstream H3K14ac-enriched exons mainly when mid-excluded (SACS7, Fig. 3d). Strikingly, 37% of the exons annotated as constitutive, meaning always included in all the mRNAs transcribed and processed from that locus, are also marked by their own and unique chromatin signature not found at alternatively spliced exons, H4K20me1 + H3K36me3 (SACS8, Figs. 2a and 3h and Supplementary Data 3), which further supports a role for chromatin in regulating exon recognition and splicing. Interestingly, using the same unbiased strategy with publicly available ChIP-seq datasets, we could also find some of these splicing-associated chromatin signatures in mouse embryonic stem cells, such as SACS4, with 57% of the studied excluded exons strongly marked by H4K20me1 + H3K79m2, which may indicate a specific and evolutionary conserved role of some of these SACS in stem cells alternative splicing (Supplementary Fig. 3g, h). We presume that with more chromatin modifications mapped in different cell types and organisms, more SACS will be identified.

To test the link between the identified chromatin signatures and alternative splicing, we then compared the levels of splicing-associated histone marks at exons that changed splicing between cell lines, with the expectation that a change in chromatin should correlate with a change in splicing.

### Splicing-associated chromatin signatures are intimately linked to alternative splicing patterns

**Fig. 4:**
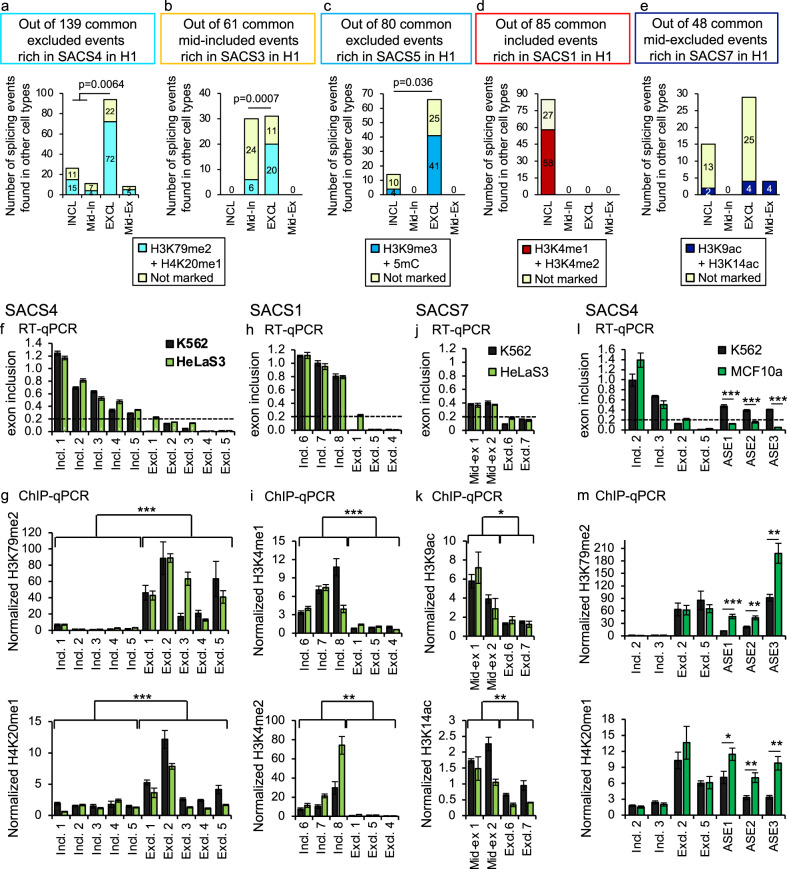
Experimental validation of SACS exons in different cell lines. **a**–**e** Number of exons marked in SACS4 (**a**), SACS3 (**b**), SACS5 (**c**), SACS1 (**d**) or SACS7 (**e**) in H1 that maintain or not the SACS enrichment when spliced in other cell lines in which the appropriate epigenomics data is available (details about the cell lines in Supplementary Data 1). Exons were grouped into included (Incl.), excluded (Excl.), mid-included (Mid-in.) or mid-excluded (Mid-ex.) depending on the pattern of splicing in the other cell lines analysed. Only alternatively spliced events co-expressed in H1 and any of the other cell lines are studied. *p*-value < 0.05 in Fisher’s exact test, two-sided. **f**–**k** Experimental validation of the results obtained in silico. H3K79me2, H4K20me1, H3K4me1, H3K4me2, H3K9ac and H3K14ac enrichment levels at alternatively spliced exons that are included (Incl.), excluded (Excl.) or mid-excluded (Mid-ex) in K562 (black) and HeLa S3 (light green) cells. In **f**, **h**, **j**, exon inclusion levels are normalised by total expression levels of the corresponding gene. Below 0.2 (highlighted with a dotted line) the exon is considered excluded. Data is depicted as the Mean ± SEM of *n* = 4 independent experiments by quantitative RT-qPCR. In **g**, **i**, **k** the enrichment levels (% input) of the studied histone marks at the alternatively spliced exon are normalised to two control regions that remain unchanged between cell types. Data is depicted as the Mean ± SEM of at least *n* = 4 independent experiments by quantitative ChIP-qPCR. **p-value < 0.01, ***p-value < 10^−5^ (*T*-Test, two-sided). **l**, **m** Same as **f**, **g**, but this time three alternatively spliced events (AS) that switch exon inclusion levels between K562 (black) and MCF10a (green) are shown. Two included and two excluded events that do not change between cell types are shown as controls. *p-value < 0.05, **p-value < 0.01 and ***p-value < 0.001 (*T*-Test, two-sided).

A splicing shift from excluded to included is not a frequent event between cell lines. In order to accumulate enough exons for a robust statistical analysis of the correlation between changes in chromatin and splicing, we collected data from nine different cell lines for which relevant transcriptomics and epigenomics data is publicly available (see Supplementary Data 1 for a list of the datasets). We first selected all the exons marked by a SACS in H1 cells that are also expressed in at least one of the cell types analysed. Then, for each cell line, we classified the exons between splicing groups and calculated the ChIP-seq coverage along the exon of the two histone marks defining the SACS. We found that for SACS4, most of the excluded events marked by H4K20me1 + H3K79me2 in H1 cells remain excluded and enriched in these histone marks in the other cell types (72/94 = 77%), whereas nearly half of the exons that shift to included/mid-included lose the SACS4 chromatin signature (Fig. 4a). Importantly, when performing the same type of analysis, but this time selecting for mid-included events marked by H4K20me1 + H4K91ac in H1 cells (SACS3), we found that 65% (20/31) of the events that shift their pattern of splicing from mid-included (in H1) to excluded (in any other cell type), also change their chromatin signature from H4K20me1 + H4K91ac to H4K20me1 + H3K79me2 (Fig. 4b). In contrast, barely 20% (6/30) of the events that maintain the same inclusion levels are enriched in H4K20me1 + H3K79me2. Similar analyses in other SACS exons, such as SACS1, SACS5 and SACS7, confirmed the change in SACS enrichment at exons that also change splicing levels between cell lines, such as SACS5 and SACS7, while exons maintaining their splicing levels conserve the same SACS, like SACS5 and SACS1(Fig. 4c–e). Even more, when looking at the overlap of SACS-marked exons between cell lines, in which epigenomics data is available such as SACS4 and SACS1 in HepG2 and K562 cells, we found percentages of spliced exons marked by a SACS comparable to H1 cells with a high overlap of SACS-marked exons between the two cell lines (70% and 83% overlap of SACS4 and SACS1-marked exons, respectively), suggesting conservation of SACS between cell lines (Supplementary Fig. 3i, j). All together, these results support a link between specific chromatin signatures and alternative splicing patterns across cell types that might be evolutionary conserved, such as SACS4 in mouse.

To validate these in silico results, we performed ChIP-qPCR and RT-qPCR in three unrelated human cell lines: tumoral hematopoietic K562, tumoral epithelial HeLa S3 and normal epithelial MCF10 cells (Fig. 4f–m). We first confirmed that excluded events are more enriched in H3K79me2 and H4K20me1 than included (SACS4, Fig. 4f, g). Of note, in some cases, H4K20me1 levels in mid-included exons are as high as in excluded, which is consistent with its enrichment in SACS3. As predicted by SACS1, H3K4me1 and H3K4me2 are significantly enriched at included exons, while H3K9ac and H3K14ac are more enriched in mid-excluded than excluded exons, as predicted by SACS7 (Fig. 4h–k). Importantly when looking at three splicing events that shift from included in K562 to excluded in MCF10a, a significant increase in H3K79me2 and H4K20me1 levels is observed only in MCF10a cells, confirming a negative correlation between enrichment of these two marks and exon inclusion levels, as predicted by our model (Fig. 4l, m).

In conclusion, we could validate in the same subset of exons and in different cell types the existence of splicing-associated chromatin signatures which enrichment levels change depending on the level of exon inclusion. This close correlation between splicing levels and the type of SACS, in which a shift in exon inclusion levels could be translated into a change in SACS enrichment (from SACS3 to SACS4 when the exon is excluded, Fig. 4b), strongly support a functional link between the chromatin and splicing machineries. Further functional analyses are necessary to prove the causality of these marks in splicing.

We next aimed to identify the properties that these chromatin-marked exons had in common and whether chromatin is playing a role in defining their pattern of splicing.

### Exons marked by a particular splicing-associated chromatin signature share genetic and functional features

**Fig. 5:**
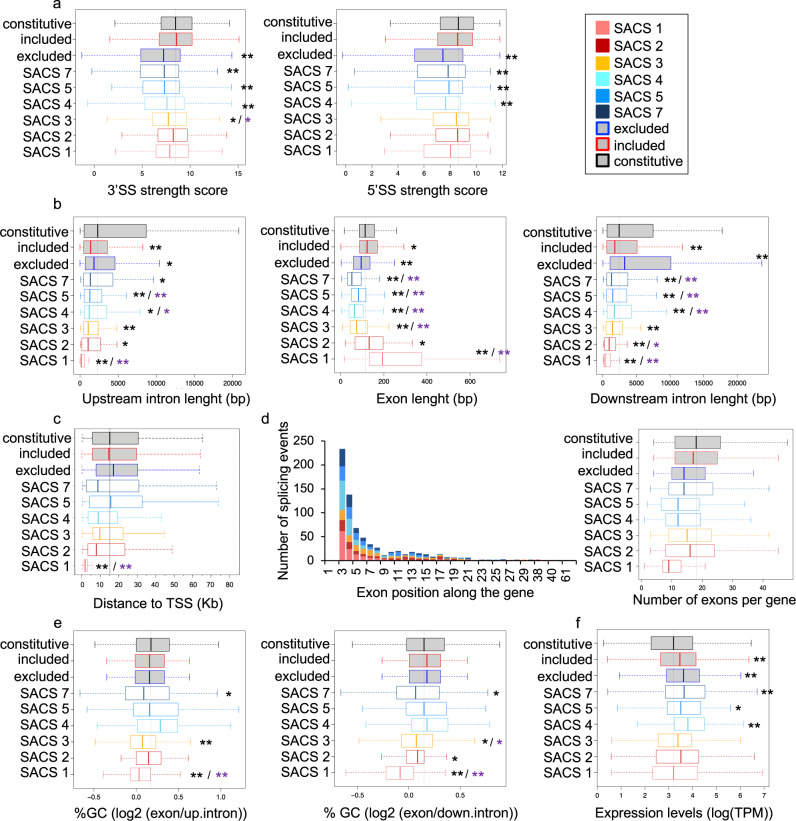
The genetic features of chromatin-marked alternatively spliced exons (SACS). **a** Box plots of the 3′ and 5′ splice site (ss) strength scores. **b** Box plots of exon and upstream and downstream intron lengths (in bp). **c** Box plot of the distance in Kb to the transcription start site (TSS). **d** Cumulative bar graph representing the number of splicing events at each exon position along the gene and box plot of the number of exons per gene. **e** Box plots of the log2 ratio of the percentage of GC content at the alternatively spliced exon respect the upstream or downstream flanking intron. **f** Box plot of the normalised gene expression levels, represented as log(TPM). Each chromatin-marked splicing group (SACS) has its own colour code as indicated in legend. Box plots are centred on the median with interquartile ranges of all the exons enriched in a particular SACS with SACS1 exons *n* = 165, SACS2 exons *n* = 142, SACS3 exons *n* = 143, SACS4 exons *n* = 152, SACS5 exons *n* = 89, SACS7 exons *n* = 139, non-marked excluded exons *n* = 600, non-marked included exons *n* = 600 and Constitutive exons *n* = 600. Constitutive exons (in grey+black) and non-marked excluded+mid-excluded (in grey+blue) and included+mid-included (in grey+red) exons are used as controls. *p-value < 0.01 and **p-value < 0.001 in Wilcoxon rank test, two-sided, compared to constitutive exons (in black) or the corresponding alternatively spliced control exons (in purple).

When looking at characteristic features shared by exons marked by a specific SACS, we found that most of the chromatin-marked exons are shorter than constitutive exons, have weaker 3′ and 5′ splice sites and are surrounded by shorter flanking introns (Fig. 5a, b), which is consistent with previous observations pointing to a role for chromatin in improving the recognition of suboptimal exons by the splicing machinery^8,21,22,25^. Interestingly, SACS1 and 2 are the only groups with longer exons than constitutive and no differences in splice site strength, suggesting a different mechanism of chromatin-mediated splicing regulation (Fig. 5a, b). Surprisingly, despite disregarding the two first exons of the alternatively spliced genes analysed, SACS1 exons were found closer to the transcriptional start site (TSS) (between 1-5 kb) than any other SACS exon (Fig. 5c). Interestingly, the position in the gene of SACS-marked exons is comparable between groups, with a preference for the third and fourth position. Moreover, there are no major differences in the number of exons per SACS-marked genes except for SACS1 that are smaller genes, which might explained the proximity to the TSS of SACS1-marked exons (Fig. 5d). Taken together with the observation that very long SACS1-marked exons have the shortest flanking introns, it suggests that SACS1-marked genes might have a very particular gene structure that could play a role in the splicing regulation. Importantly, the exons differentially enriched in methyl DNA levels (SACS2 and SACS5) do not show significant changes in their GC content compared to non-marked alternatively spliced exons, arguing against 5mC changes just because of differences in the percentage of GC between exons (Fig. 5e). Finally, although some alternatively spliced genes, marked or not by a SACS, have different total gene expression levels respect constitutively spliced genes, there are no major differences between all the alternatively spliced genes with or without a SACS exon, ruling out a transcription effect on these splicing-associated chromatin signatures (Fig. 5f).

Despite a certain degree of gene overlap between SACS groups because some alternatively spliced genes have more than one splicing event marked by a SACS, gene ontology analysis revealed that each SACS group is enriched in distinctive biological processes not found in the other groups, suggesting that chromatin might differentially mark exons sharing common functional and/or regulatory pathways (Supplementary Fig. 4). For instance, genes with excluded exons marked by SACS4 are strongly enriched in biological terms related to gene expression and RNA regulation. While mid-included events marked by SACS3 are related to adherent junction, actin-filament assembly and Golgi vesicle budding; and included events marked by SACS2 are strongly related to cancer (Supplementary Fig. 4). Importantly, GO analysis using unique gene lists revealed similar results, suggesting that overlapping genes are not responsible for the characteristic biological terms found at each SACS (Supplementary Fig. 5). Interestingly, when looking at the gene ontology of the genes with excluded exons differentially marked by H4K20me1 + H3K79me2 in mice (SACS4), many terms were found in common with the ones found in human cells (Supplementary Fig. 4), suggesting a conserved functional chromatin marking of alternatively spliced exons involved in specific regulatory pathways. We thus propose that SACSs can play a role in coordinating efficient and prompt splicing responses by just modifying key histone modifications at key regulatory genes, instead of inducing global changes in the expression patterns of more pleiotropic splicing regulators.

**Fig. 6:**
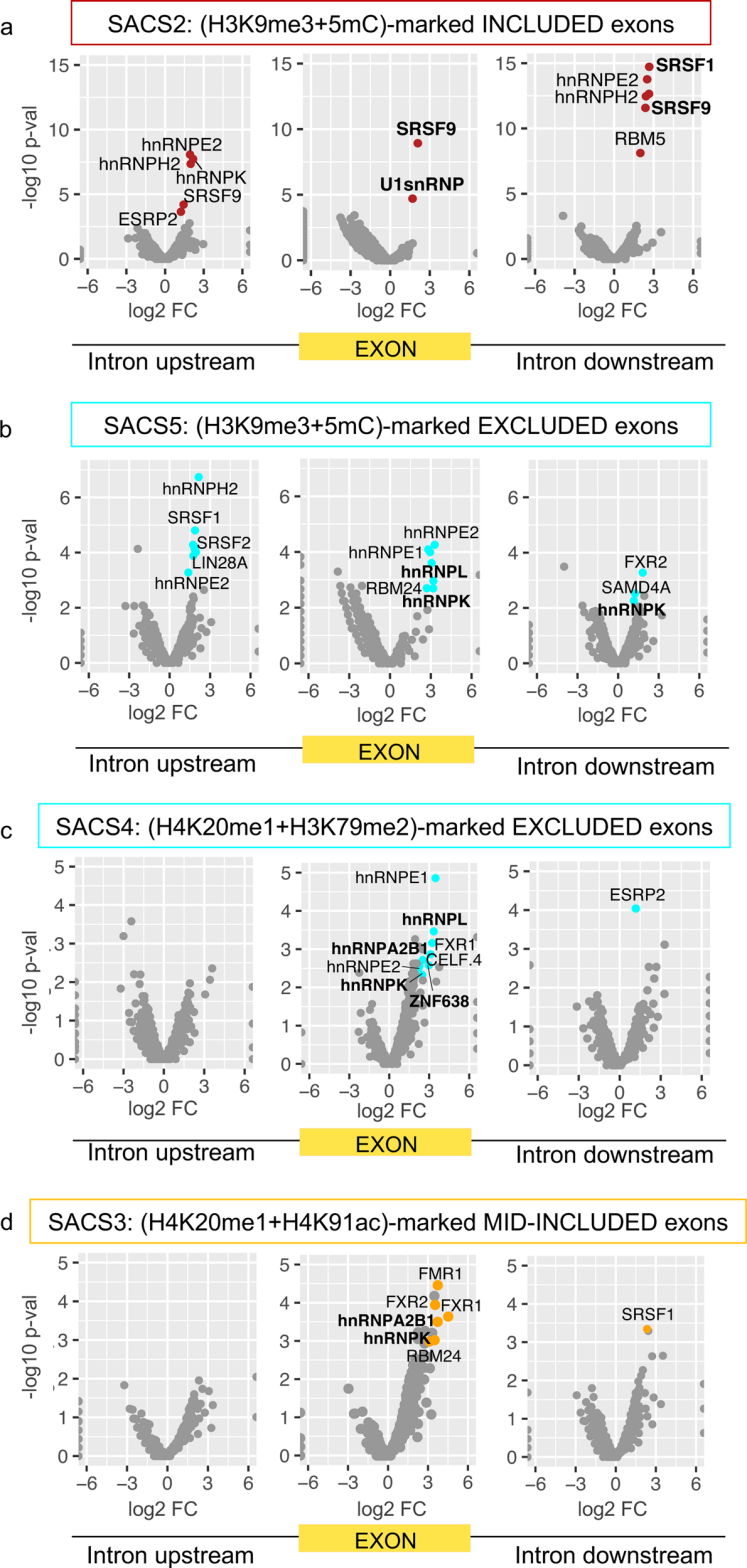
SACS are defined by specific RNA-binding protein (RBP) motifs. **a**–**d** Volcano plots of the scanned RBP motifs and 5mers in the upstream intron (left), chromatin-marked exon (middle) and downstream intron (right) for **a** included H3K9me3 + 5mC-marked exons (SACS2), **b** excluded H3K9me3 + 5mC-marked exons (SACS5), **c** excluded H4K20me1 + H3K79me2-marked exons (SACS4) and **d** mid-included H4K20me1 + H4K91ac-marked exons (SACS3). Coloured dots correspond to motifs with adjusted *p*-value < 0.01 and Benjamini and Hochberg false discovery rate FDR < 0.05. *X* axis represents the log2 fold enrichment (FC) of each motif compared to non-marked alternatively spliced events sequences. *Y* axis represents the −log10 adjusted *p*-value of the enrichment. FDR and associated adjusted *p*-value were calculated from *n* = 152 H4K20me1 + H3K79me2 excluded exons, *n* = 89 H3K9me3 + 5mC excluded, *n* = 143 H4K20me1 + H4K91ac mid-included exons, *n* = 142 H3K9me3 + 5mC included exons, *n* = 600 non chromatin-marked excluded exons and *n* = 600 non chromatin-marked included exons.

Following these lines, if chromatin-marked exons shared common regulatory pathways, it should be reflected by the presence of common RNA motifs. To address this hypothesis, we looked into the presence of SACS-specific RNA binding sites, responsible for the recruitment of specific splicing regulators to the pre-mRNA. For each group of chromatin-marked splicing events, we scanned for known RNA motifs from the CISBP-RNA database^40^ that were significantly more enriched at the chromatin-marked exons, or flanking intronic sequences, compared to alternatively spliced exons not marked by a chromatin signature. As expected, we found characteristic RNA motifs in 4 of the 7 SACS identified (Fig. 6). Some of them are common between more than one SACS, such as hnRNPK and hnRNPL (SACS3, 4 and 5 - Fig. 6b, c, d). Whereas others are unique to a specific group, such as U1 snRNP and SRSF9 (SACS2 - Fig. 6a), or the Zn-Finger protein ZNF638 (SACS4 - Fig. 6c). Interestingly, hnRNPK and hnRNPL have been shown to directly interact with histone methyltransferases^41,42^, and ZNF638 is a transcriptional cofactor shown to regulate splicing, during adipocyte differentiation, by directly interacting with the splicing machinery^43,44^, which supports a functional link between chromatin and the recruitment of specific splicing regulators to the pre-mRNA.

We next delved deeper into how these splicing-associated chromatin signatures might impact the splicing machinery.

### Splicing-associated chromatin signatures can impact the recruitment of RNA binding proteins to the pre-mRNA

**Fig. 7:**
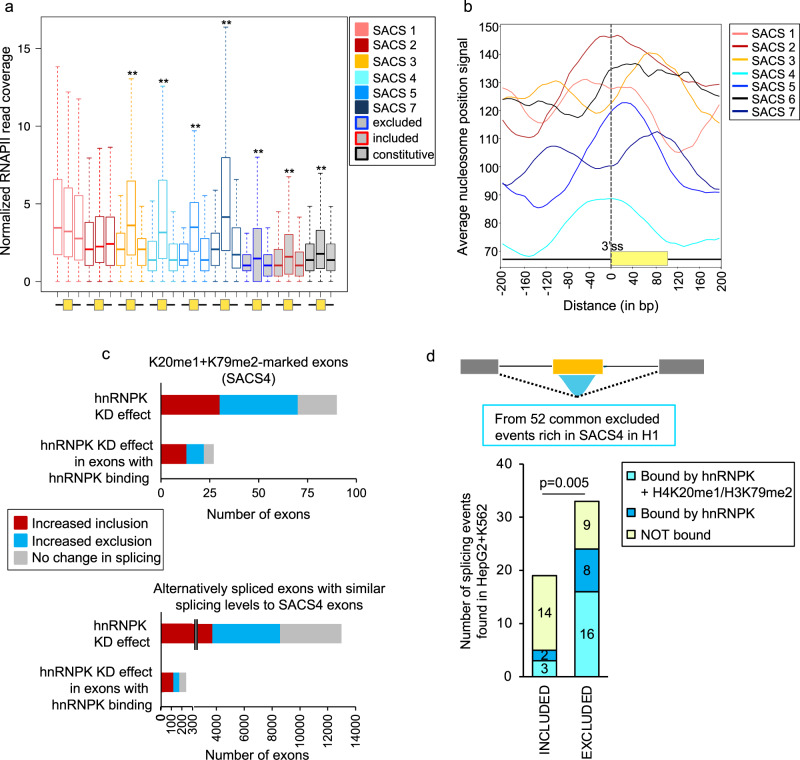
SACS can impact RNA polymerase II distribution and recruitment of splicing factors. **a** Box plot centred on the median with interquartile ranges of the normalised RNA polymerase II (RNAPII) reads coverage over the upstream intron, exon and downstream intron for the chromatin-marked exons. Constitutive and non-marked excluded and included exons are used as controls (shaded in grey). RNA polymerase II is more enriched at exons than introns in all conditions except for H3K4me1 + H3K4me2 (SACS1) and H3K9me3 + 5mC (SACS2) included exons. **p-value < 0.01 at exons compared to flanking introns in Wilcoxon rank test, two-sided. SACS1 exons *n* = 165, SACS2 exons *n* = 142, SACS3 exons *n* = 143, SACS4 exons *n* = 152, SACS5 exons *n* = 89, SACS7 exons *n* = 139, non-marked excluded exons *n* = 600, non-marked included exons *n* = 600 and Constitutive exons *n* = 600. **b** Average nucleosome occupancy signal ±200 bp the exon start at the 3’ss for each SACS group. **c** Splicing effect on alternatively spliced exons upon hnRNPK knockdown, using available data from GM19238 cells. Only genes expressed both in H1 and GM19238 cells were studied. The number of hnRNPK-dependent events with hnRNPK binding evidence, using publicly available eCLIP data in K562 and HepG2 cells, is also shown. Exons that are more included upon hnRNPK knockdown are shown in red, more excluded are shown in blue and not affected are shown in grey. **d** hnRNPK binding and enrichment of H4K20me1 + H3K79me2 levels at alternatively spliced exons shifting splicing patterns in different cell lines. Using available eCLIP and ChIP-seq data in K562 and HepG2 cells, we found that from 52 excluded exons rich in H4K20me1 + H3K79me2 in H1 hESC, 33 remained excluded and 19 switched to included in K562 or HepG2. Excluded events were more co-enriched in H4K20me1 + H3K79me2 than included (Fisher’s exact test, two-sided, *p*-value < 0.05) and most of the (H4K20me1 + H3K79me2)-rich excluded events were bound by hnRNPK (Fisher’s exact test, two-sided, *p*-value < 0.05), supporting a model in which a specific chromatin signature can favour the recruitment of a splicing regulator to the pre-mRNA.

Chromatin has been shown to regulate splicing via two models, the RNA polymerase II kinetic model and the chromatin-adaptor recruitment model. To gain insights into how the identified chromatin signatures might regulate splicing, we first looked into the distribution of RNA polymerase II along SACS-marked exons and their flanking intronic regions, using constitutively spliced and non-marked alternatively spliced exons as controls (Fig. 7a). ChIP-seq and NET-seq studies have shown that RNA polymerase II occupancy is higher at exons with well-defined nucleosomes positioned at the exon^45,46^. These nucleosomes act as roadblocks that modulate RNA polymerase II elongation rate by inducing pausing at specific sites to increase the window of time for splicing regulators to be recruited to weak splice sites. Importantly, a shift in nucleosome positioning can be sufficient to induce a change in RNA polymerase II pausing and alternative splicing^17,20^. When studying RNA polymerase II occupancy at exons differentially marked by a SACS, we observed that RNA polymerase II levels were significantly higher at exons compared to flanking introns, except for SACS1 and SACS2, in which there were similar enrichment levels at the exon and flanking introns (Fig. 7a). SACS1 and SACS2 are precisely the two groups in which we observe an enrichment of the chromatin marks upstream and downstream the exon, respectively, suggesting the presence of a nucleosome at these intronic regions that could impact RNA polymerase II pausing. Estimation of the average nucleosome positioning along SACS-marked exons, using available MNAse data in H1 hESC from the NucMap portal, confirmed the existence of a nucleosome upstream SACS1 exons (Fig. 7b). Moreover, the promoter-proximity of SACS1 exons might also influence RNA polymerase II pausing at these sites, which would affect exon inclusion based on the kinetic model^11,37^. However, nucleosomes were not found positioned downstream SACS2 exons, but rather upstream right at the intron-exon junction, which could still impact RNA polymerase elongation rate (Fig. 7b). Interestingly, even though nucleosomes are also at the intron-exon junction at SACS4 exons, we did not observe an impact on RNA polymerase II distribution, suggesting that a shift in the nucleosome positioning is not sufficient and might need to be complemented with the enrichment of a specific chromatin signature to impact RNA polymerase II kinetics, such as H3K9me3 + 5mC downstream the exon in the case of SACS2. In support of an elongation rate mediated effect of SACS1 and SACS2 in splicing, exons affected by elongation rate mutants are longer than non-affected exons^47^, which is consistent with SACS1 and SACS2 exons having a bigger exon size than the rest (Fig. 5b).

Finally, regarding H4K20me1-marked exons (SACS3 and 4), which are two SACS with clear enriched RNA binding motifs, we found common RNA motifs (FXR1, hnRNPA2B1 and hnRNPK) between the two groups (Fig. 6c, d). Since these two groups share the same histone mark (H4K20me1), but have two opposite splicing outcomes (mid-included and excluded), we suggest a model in which the enrichment of a chromatin signature might impact the recruitment of a RNA binding protein to the regulated exon, thereby modulating the final splicing outcome. To test this hypothesis, we took advantage of available hnRNPK knockdown and eCLIP data from different cell lines to correlate enrichment of SACS4 with functional dependence on the splicing repressor hnRNPK (Fig. 7c, d). We found that 78% of SACS4-marked exons are dependent on hnRNPK levels, amongst which 81% have evidence of binding by the splicing repressor. Whereas 66% of alternatively spliced exons, marked or not by chromatin modifications and with similar splicing levels to SACS4-marked exons, are affected by hnRNPK knockdown (Fig. 7c). Even more, when comparing H4K20me1 + H3K79me2 and hnRNPK levels between cell lines in which SACS4-marked exons change splicing patterns, we found that 48.5% (16/33) of the exons that stay excluded between cell lines keep the SACS4 signature, and are bound by hnRNPK. Whereas 16% (3/19) of the exons that shift to included in HepG2 or K562 cells lines keep these signatures (Fig. 7d), further confirming a preferential binding of hnRNPK to H4K20me1 + H3K79me2-rich exons. It is important to note that several of the RNA motifs found to be enriched along these chromatin-marked exons, such as hnRNPL, hnRNPK and hnRNPA2B1, have been reported to directly interact with specific chromatin regulators or to be associated with the chromatin fraction in mass spectrometry analyses^41,42,48^, supporting the idea that different combinations of histone marks can influence the recruitment of chromatin and splicing regulators to the pre-mRNA via protein-protein interactions.

## Discussion

Since the discovery of co-transcriptional splicing, there have been many attempts to assess, at a genome-wide level, the impact of histone modifications in alternative splicing. Using a supervised machine learning algorithm, we found that 34% of the alternatively spliced cassette exons expressed in human embryonic stem cells (hESC) are differentially marked by specific combinations of 11 chromatin modifications, including DNA methylation. We show that these splicing-associated chromatin marks are highly localised along the exon and in a combinatorial way, creating specific chromatin signatures that we called SACS (Splicing-Associated Chromatin Signature). Even more, exons belonging to a specific SACS share common regulatory features, such as weaker RNA splice sites, different exon lengths and enrichment of specific RNA binding sites. Moreover, genes with SACS-marked exons are enriched in distinctive gene ontology biological terms, which all together points to a coordinated regulation of specific subsets of exons with specific functions in the cell.

The study of the impact of these SACSs in splicing revealed two potential mechanisms of splicing regulation. First, we observed that the specific positioning of the chromatin marks upstream or downstream the regulated exon could impact RNA polymerase II distribution along SACS-marked exons, like in SACS1 and SACS2. It has been previously reported that a change in nucleosome positioning along the exon can impact RNA polymerase II elongation rate and thus splicing via the kinetic model^17,20,37^. We did observe a change in RNA polymerase II distribution at SACS-marked exons with a shift in nucleosome positioning upstream the regulated exon (SACS1 and SACS2). However, since SACS4 exons did not show differences in RNA polymerase II distribution, despite a shift in nucleosome positioning similar to SACS2, and we could not recapitulate in SACS2 the expected nucleosome positioning downstream the exon, we propose an alternative mechanism of modulation of RNA polymerase II elongation rate. It has actually been shown that histone marks can also impact RNA polymerase II elongation rate by recruiting chromatin binding proteins that slow down the RNA polymerase II, such as HP1 binding to H3K9me3-rich chromatin, which is a SACS2 mark^18,29^. Position-dependent enrichment of specific chromatin modifications can thus impact RNA polymerase II pausing via nucleosome positioning (SACS1) and/or recruitment of chromatin binding proteins (SACS2) to specific chromatin marks, which in turn can impact splicing. Of note, three-dimensional (3D) chromatin interactions between exons and promoters, which can impact RNA polymerase II elongation rate, have recently been shown to play a role in the regulation of alternative splicing^11^. It is thus possible that changes in 3D chromatin structure might also be involved in some of the SACS-mediated mechanisms of splicing regulation, which is an aspect we are currently addressing in the laboratory. Finally, the enrichment of specific chromatin regulators along the exon can also create docking sites for recruitment of splicing regulators, such as hnRNPK, to their RNA binding sites via protein-protein interactions, which is known as the recruitment model^26^. The coupling of specific combinations of histone marks with specific splicing regulators, which RNA binding motifs are significantly enriched at these SACS-marked exons, would thus increase the specificity and dynamics of splicing regulation. By just changing exon-specific histone marks, the recruitment of a splicing regulator could be immediately affected without the need to impact its transcriptional levels, increasing like this the plasticity of the splicing machinery. In support of these SACS-mediated regulation of splicing, we found that exons changing splicing levels between cell lines, also changed of SACS levels, while non-changing alternatively spliced exons conserved their characteristic SACS regardless of the cell line, supporting a role in maintaining specific splicing patterns.

Even though the causative role in splicing of some of the chromatin marks identified in this study, such as H3K4me3, H3K27me3, H3K9me3 and DNA methylation, has already been proved in some model genes^18,26,27,29^, the global impact at a genome-wide level still remains elusive. Interestingly, most of the studies agree that it is a small subset, rather than the vast majority, of regulated exons that are sensitive to chromatin. In our study, 34% of all the cassette exons analysed in H1 embryonic stem cells are marked by some type of chromatin modification. When comparing this small subset of exons to all the regulated exons in the human genome (~70,000 exons), the percentage drops to ~1,25%. Despite this percentage is largely underestimated, since we are comparing the exons well expressed in a cell line to the whole transcriptome, it still reflects the high specificity of this chromatin-mediated mechanism of splicing regulation in which a restraint number of exons, sharing common regulatory and/or functional features, are coordinated by changes in specific histone marks. For instance, when correlating changes in splicing with changes in chromatin in 34 normal and blood cancer cell lines, 35% of the alternatively spliced exons analysed were enriched in H3K79me2. A chromatin-mediated change in the alternative splicing of these H3K79me2-marked exons, by knocking-down the H3K79 methyltransferase Dot1L, significantly reduced cell proliferation and tumour progression in two of these leukaemia cancer cell lines, highlighting the coordinated regulation of cancer-related splicing events by H3K79me2^35^. In stem cell differentiation, alternatively spliced genes with exons marked by H3K36me3, H3K27ac and H3K8ac shared common gene ontology terms related to stemness signatures, G2/M cell-cycle progression and DNA damage response, suggesting again that chromatin might coordinate the regulation of specific splicing-related pathways important for cell differentiation^36^. Nevertheless, more functional studies are necessary to properly assess the causal link and biological impact of chromatin in cell-specific splicing. Of particular interest will be to study such a link in a disease context, as a potential target for novel therapeutic treatments, and in highly dynamic situations, such as in response to external stimuli. Indeed, in plants, the chromatin remodeler ZmCHB101 has been shown to impact alternative splicing in response to osmotic stress^49^. Furthermore, reduction in H3K36me3 or H3K36me-binding proteins from the MRG15 family, which has been shown by our group to induce recruitment of splicing regulators to the pre-mRNA^26^, affects splicing of genes important for Arabidopsis flowering in response to temperature^50,51^, supporting a functional link between chromatin and splicing in response to environmental stimuli.

Another important result is that splicing-associated chromatin signatures (SACS) are not only marking well included and excluded exons, but also exons with intermediary inclusion levels, such as SACS3 and SACS7, suggesting that chromatin could play a role in regulating the number of transcripts that include or not a particular exon in a cell population or at the single cell level, thus creating a protein diversity that could be important for cell adaptability or response to certain external stimuli. In support of this hypothesis, a recent study using single cell deep sequencing transcriptomics has shown that there can be more than one splicing isoform per gene in a single cell, and that 8% of all the alternatively spliced exons analysed are also differentially marked by DNA methylation^52^. Importantly, this methylation-splicing association was stronger when looking at cells individually, in contrast to bulk data using mean values, pointing to an underestimation of the impact of chromatin in splicing decisions when using cell population data, such as in our study^52^. Taken together, these results point to a role for chromatin modifications in regulating the cell-to-cell and transcript-to-transcript splicing variability necessary to create a splicing diversity at the single cell level that is important to increase the cell’s adaptability to rapidly changing conditions.

In conclusion, we have identified 11 chromatin modifications that differentially mark alternatively spliced exons in a highly localised and combinatorial way. A shift in exon inclusion levels between different cell types correlates well with changes in the enrichment levels of the histone marks and splicing regulators predicted by our model, further supporting a role for chromatin in regulating the recruitment of the splicing machinery to the pre-mRNA. The fact that chromatin usually marks shorter exons with longer flanking introns and weaker splice sites supports a role for chromatin in improving exon definition and the recognition of the exon by the splicing machinery^22,48^. However, in this study we find that exons are differentially marked by specific combinations of chromatin modifications depending on the level of exon inclusion, suggesting that it is not just about exon recognition, but also about the level of splicing diversity needed by the cell. Of note, even though the whole study is based on embryonic H1 stem cells and foetal IMR90 cells, we are validating these SACS in other adult cells, such as MCF10a, K562 and HeLa, suggesting that this is not something inherent to developmental stages, but it is conserved in adult cells. Further studies, particularly at the single cell level and in physiologically relevant model systems, such as disease, will be essential to further understand the importance of chromatin in splicing and cell biology.

## Methods

### Cell lines and culture

K562 human immortalised chronic myelogenous leukaemia bone marrow cells were grown in IMDM + Glutamax-I (Gibco), supplemented with 10% fetal bovine serum (Sigma) and 1x Pen./Strep. (Sigma). Human cervix adenocarcinoma HeLa S3 cells were cultured in Ham’s F-12 nutrient mix with 2 mM l-Glutamine (Gibco), supplemented with 10% fetal bovine serum (Sigma) and 1x Pen./Strep. (Sigma). Human mammary epithelial MCF10a cells were cultured in DMEM/F-12 (Sigma) supplemented with 5% horse serum (Thermofisher), 20 ng/ml EGF (Sigma), 0.5 µg/ml hydrocortisone (Sigma), 100 ng/ml cholera toxin (Sigma), 10 µg/ml insulin (Sigma), 2 mM l-Glutamine (Thermofisher) and 1x Pen./Strep. (Sigma)^26^. All cells were grown at 37^o^C, 5% CO2, and were regularly tested for mycoplasma presence.

### RNA extraction and real-time RT-qPCR

RNA is extracted from cell pellets using Trizol (Life technologies) and then cleaned-up using the GeneJET RNA purification kit (Thermofisher), following the manufacturer’s instructions. 500 ng of total RNA is retrotranscribed into cDNAs using the Transcriptor First Strand cDNA Synthesis kit (Roche, 04897030001). Quantitative qPCRs were performed on the Bio-Rad CFX-96 Real-Time PCR System using iTaq Universal Sybr green Supermix (Bio-Rad)^26^. Four biological replicates were performed per gene and cell type. Data was plotted as mean ± S.E.M. Exon-inclusion levels are calculated by normalising the expression levels of the regulated exon with total expression levels of the gene calculated from constitutive exons. See Supplementary Data 5 for a list of primers used.

### Chromatin Immunoprecipitation

K562, HeLa or MCF10a cells are cross-linked with 1% formaldehyde (Thermo Scientific) in DMEM/F-12 (Sigma) for 2 min. The reaction is quenched by the addition of glycine (Sigma) at a final concentration of 125 mM for 5 min. Cells are then washed twice with PBS, and resuspended in 1 ml of lysis buffer A [50 mM Hepes (Sigma, H3375) pH 7.5; 140 mM NaCl (Sigma, S5150); 1 mM EDTA (Gibco, 15575-038); 10% Glycerol; 0.5% IGEPAL CA-630 (Sigma, I3021); 0.25% Triton X-100 (Sigma, X100); 1x Complete protease inhibitor mixture (Roche, 4693159001), 200 nM PMSF (Sigma, P7626)]. After 10 min on ice, the cells are pelleted and resuspended in 1 ml of lysis buffer B [10 mM Tris-HCl (Sigma, T2663) pH 8.0; 200 mM NaCl (Sigma, S5150); 1 mM EDTA (Gibco, 15575-038); 0.5 mM EGTA (Bioworld, 40520008-2); 1x protease inhibitors (Roche, 4693159001); 200 nM PMSF (Sigma, P7626)]. After 10 min at room temperature, cells are sonicated in lysis buffer C [10 mM Tris-HCl (Sigma, T2663) pH 8.0; 100 mM NaCl (Sigma, S5150); 1 mM EDTA (Gibco, 15575-038); 0.5 mM EGTA (Bioworld, 40520008-2); 0.1% sodium deoxycholate (Sigma, 30970); 0.5% N-lauroylsarcosine (MP, Biomedicals, 190110); 1x protease inhibitors (Roche, 4693159001); 200 nM PMSF (Sigma, P7626)] using Diagenode Bioruptor for 12 cycles (30 sec ON; 50 sec OFF) to obtain ~200–500 bp fragments. Cell debris are pre-cleared by centrifugation at 21,000 xg for 20 min, and 8 μg of chromatin is incubated with 1 μg of either anti-H3K79me2 (Abcam, ab3594), anti-H4K20me1 (Abcam, ab9051), anti-H3K4me1 (Abcam, ab8895), anti-H3K4me2 (Abcam, ab7766), anti-H3K9ac (Abcam, ab4441) and anti-H3K14ac (Abcam, ab52946) antibodies overnight at 4 °C. Protein G-conjugated magnetic beads (Invitrogen, 10009D) are added the next day for 2 h. Subsequent washing and reverse cross-linking are performed^26^. Quantitative qPCRs were performed on the Bio-Rad CFX-96 Real-Time PCR System using iTaq Universal Sybr green Supermix (Bio-Rad). At least four biological replicates were performed for each histone mark and cell line (see Source Data file for details). Data was plotted as mean ± S.E.M. ChIP enrichment for a primer-set was evaluated using the percentage of input normalised to two control primer-set, used as a reference to reduce technical and cell-to-cell variability. See Supplementary Data 5 for a list of the primers used.

### Analysis of high-throughput sequencing datasets

Available paired-end RNA-seq data was downloaded from the ENCODE portal (https://www.encodeproject.org/), which compiles both ENCODE and ROADMAP data. Percent of inclusion (PSI) of alternatively spliced exons was calculated using the raw fastq files, in which reads were filtered following the ENCODE guidelines, for human H1 hESC, K562, IMR90, A549, Gm12878, GM19238, HEK293, HelaS3, HepG2 and MCF7 cell lines and mouse mESC files. RNA-seq from the nuclear fraction was used for downstream analysis if total RNA-seq was not available. Data was aligned in hg19 genome with STAR 2.3.0e^53^ with parameters;–runThreadN 4–outFilterMismatchNmax 2–clip3pAdapterSeq AAAAAA–clip3pAdapterMMp 0 –outSJfilterReads Unique –alignSJDBoverhangMin 3 –alignSJoverhangMin 5–outSJfilterOverhangMin 30 12 12 12–outSJfilterCountUniqueMin 5 1 1 1–outSJfilterIntronMaxVsReadN 50000 100000 200000–sjdbScore 2–outFilterType BySJout–outSAMattributes All–seedSearchStartLmax 50, using an overhang of 99nt for long RNAseq reads and a custom database of exon–intron junction annotations from https://github.com/nellore/intropolis. All the exons and introns were extracted from BioMart using Ensembl72 annotations. Alternatively spliced cassette exons, in which an alternatively spliced exon is flanked by two constitutive exons, were extracted. First and second exons were excluded from the analysis to avoid a chromatin effect from the transcription start sites. Using the Ensembl biotype term, we also discarded from the final dataset all the exons not labelled as protein coding or noncoding RNA. We considered as constitutive exons all the exons annotated with the constitutive exon term in Ensembl72 that were coming from the same transcripts as the selected alternatively splice exons and were included at a PSI > 95% in more than 75% of the 10 cell lines analysed. For each cell line the SJ.out.tab file from STAR output was filtered to recover all the exon-intron junctions present at least with a count of 5 reads. We extracted the number of reads at all the exon–intron (for inclusion) and exon–exon (for exclusion) junctions from the final exon triplets dataset. Final PSI was calculated as a ratio of the number of reads including the exon, divided by the sum of the exclusion and inclusion reads. For the inclusion reads we considered the average value, since we expect reads coming from the acceptor and donor sites. Based on the cumulative distribution of PSI in H1 and IMR90 cell lines, four splicing groups were created: excluded (0% < PSI < 20%), mid-excluded (20% < PSI < 40%), mid-included (40% < PSI < 80%) and included (80% < PSI < 100%).

Regarding gene expression, it was calculated with Salmon 0.8.2^54^, first we quantified the transcriptome for each cell line, using the Ensembl72 transcripts fasta file, and then selected for genes with TPM values ≥10 to discard lowly expressed gene. When comparing different cell lines, alternatively spliced genes with different expression levels were excluded to avoid confounding transcriptional effects.

ChIP-Seq and MeDip-seq data were obtained from the ENCODE portal (https://www.encodeproject.org/), which compiles both ENCODE and ROADMAP data. Reads were mapped to the reference genome hg19 using Bowtie^54^, keeping the best unique matches, with at most two mismatches to the reference (−v 2 –best –strata -m 1). Reads were extended to 200 nt in the 5′ to 3′ direction using Pyicos^55^. Then using BedTools^56^, we removed repetitive reads overlapping centromeres, gaps, satellites and pericentromeric regions. For each sample, we used Pyicos to build clusters with overlapping reads along the genome, discarding single-read clusters. In order to avoid the usage of clusters that are possibly part of the background signal (and not of the real ChIP-Seq signal), we used input samples (when available) for the peak calling normalisation to identify clusters that are significantly above input values^19^.

### Feature selection for the Random Forest classifier

Random Forest classifier is an ensemble method in which classification is performed by voting of multiple unbiased weak classifiers, the decision trees. These trees are independently developed on different bagging samples of the training set. The importance measure of a feature is obtained as the loss of accuracy of classification caused by the random permutation of the feature values between the objects to classify. It is computed separately for all trees in the forest which use a given feature for classification. Then the average and standard deviation of the accuracy loss are computed.

The features we used to classify splicing events into different splicing groups were based on the read coverage of each ChIP-seq/MeDip-seq sample around the 3′ (exon start) and 5′ (exon end) splice sites (SS) of alternatively spliced exons in H1hesc and IMR90, using Ensembl72 annotation (hg19). Only exons with enough RNA-seq coverage around the splice junctions were used for assessment of the percentage of inclusion (PSI) and distribution into one of the 4 splicing groups based on splicing levels. First and second exons were discarded to avoid strong promoter effects. For each ChIP-seq/MeDip-seq sample, we calculated the normalised read density, using an approach similar to RPKM (reads per kilobase per million of mapped reads). We used BedTools bedcoverage to get the read counts 100 nt upstream and 100 nt downstream the 5′SS and 3′SS region of each of the selected exons. Read count was then normalised to region length. Maxent was used to calculate the splice site strength scores of each alternative exon. Splice site strength scores were used as a positive control of an informative feature capable of classifying included from excluded exons^57^. Then, we built a table of alternative exon events (rows) and epigenetic features (columns) for each of the four pre-defined splicing groups (excluded, mid-excluded, mid-included and included) for both H1hesc and IMR90, independently. As a classifier, we used the R package Boruta 6.0.0^38^. Boruta has the particularity of adding randomness to the system, and collecting results from the ensemble of randomised samples to reduce the misleading impact of random fluctuations and correlations. This randomness is added by the creation of “shadow features”, meaning that the classifier adds a copy of the original feature but with randomised values. The importance score of each feature is compared to the highest importance of a shadow (which reflects randomness). In this way, the features showing higher scores are given a hit at each iteration. Importantly, at each iteration, new shadow features are created by re-shuffling the values of the original feature at each exon, which creates more randomness. The final selected features will be the ones, that after 10,000 iterations, have outperformed the best shadows by accumulating hits, and thus are significantly important regardless of stochasticity of the random forest classifier or sensitivity to the presence of non-important attributes in the information system. In order to retrieve the most informative features from each of the 4 pre-defined splicing groups, we run Boruta as a binary classifier for all the pairwise comparisons between excluded, mid-excluded, mid-included and included splicing groups. For each of the runs, we selected randomly same number of events per splicing group. Boruta was run with the following parameters, mcAdj = TRUE, maxRuns = 100000, getImp = getImpRfZ, dooTrace = 2, ntree = 100000, for each of the pairwise comparisons between the four defined splicing groups. This resulted in 6 lists of ranked features per cell (Supplementary Data 2). We used this approach in H1hesc and IMR90 separately. We then, selected the significant chromatin features common in both cell lines that consisted in 15 chromatin modifications. As expected splice site strengths ranked amongst the most informative features in all the binary comparisons. As a control, we randomly shuffled exons inclusion levels into different splicing groups and re-ran Boruta with the same parameters. No significant features were obtained (Supplementary Data 2 and Supplementary Fig. 1).

### Association of a splicing group with a specific chromatin signature (SACS)

To test the co-enrichment by pairs of different histone and DNA marks at alternatively spliced exons, we looked for overlapping of ChIP-seq and MeDIP-seq reads along the alternatively spliced exons and flanking upstream (UP) and downstream (DOWN) 200-nt intronic regions by running version 0.8.1 of the script Block Bootstrap and Segmentation method^58^ with parameters -*r* 0.1 -n 10,000, where *r* is the fraction of each region in each sample and *n* is the number of bootstrap samples used. As input data, we used the 14 histone marks and DNA methylation levels, selected from the Random Forest classifier, that were above background and overlapping any of the three selected regions along alternatively spliced exons. With this method, we calculated a *z*-score corresponding to the number of standard deviations of the observed overlap compared to the random expected one. We then looked for significant reciprocal associations, where one feature was associated to a second feature in a specific region, and vice versa. If the reciprocal associations were enriched at a specific position in more than one splicing group, they were discarded as they were not considered unique for a specific position and splicing group. We obtained seven unique pairs of chromatin modifications features specifically associated to a region (upstream, exon body or downstream) for a specific splicing group (excluded, mid-excluded, mid-included, included) (Supplementary Data 3). As controls, we used constitutive exons that belonged to the same genes as the chromatin-marked exons, but did not have an enrichment of the chromatin pairs found by our approach. For consistency, we only used a random comparable number of control exons for all the comparisons by selecting 600 random constitutive. To rule out overfitting, we randomly shuffled inclusion levels and splicing groups labels and repeated the analysis. No significant unique associations were obtained when using randomised inclusion levels (Supplementary Data 3).

### RNA binding motif analysis

RNA-binding protein motif enrichments were calculated on the exonic and intronic regions of each chromatin-marked event. We used a maximum intronic flank of 250 nt upstream and downstream from each chromatin-marked event, removing 9 nt at donor site and 30 nt at the acceptor site to avoid branch point (BP), splice site (SS) and polypyrimidine tract (PPT) signals^59^. Introns smaller than 60 bp were also discarded. As a control, we retrieved intronic and exonic regions from a set of excluded and included exons that do not overlap with histone nor 5mC signal. The enrichment of 5mers and RNA motif matches from RNA compete (CISBP-RNA database)^40^ were calculated applying the motif enrichment method from^60^. Briefly, for each chromatin-marked and control event, the number of times each motif appeared in each sequence was calculated. The expected density of a motif was calculated as the ratio between the total number of occurrences over the total number of sequences at control events. For each motif, an odds ratio (motif score) was obtained. Statistically over-represented motifs were selected based on the Benjamini and Hochberg false discovery rate multiple test corrected p-value (BH-FDR < 0.05).

### Analysis of hnRNPK knockdown and eCLIP publicly available data

Illumina RNA-seq data of hnRNPK-downregulated lymphoblastoid GM19238 cells using small interfering siRNAs (GSE52834: SRR1040861, SRR1040862 and SRR1040863) was studied using VasTools^61^ to identify all the exons that were affected, or not, by hnRNPK knockdown compared to control siRNAs. The exons that were also expressed in H1 cells were kept as potential hnRNPK-dependent exons for further analysis. eCLIP processed bed files from HepG2 and K562 cell lines were downloaded from the ENCODE data portal using the hg19 genome assembly. Exonic and intronic regions of the selected chromatin marked events were overlapped with BedTools intersect^56^. Then, we counted the number of occurrences and applied a Fisher exact test to measure the difference between the different groups.

### Gene ontology enrichment analysis

To identify the biological processes enriched at each group of chromatin-marked exons, we run the Enrichr R package^62^ for KEGG 2019 Human pathways and GO Biological process 2018. Enrichr uses a Fisher’s exact test (*p*) and a combined score (*c*), in which the p-value (*p*) is combined with the *z*-score (*z*) of the deviation from the expected rank (*c* = log(*p*)·*zc* = log*p·z*). GO terms were selected based on this *p*-value (*p* < 0.01) and the combined score. The GO analysis was performed with the genes with a SACS-marked exon, regardless of gene overlap between SACS groups. For most of the SACS, there is less than 20% gene overlap, and less than 8% of splicing overlap, which suggests that the gene overlap is mostly the consequence of having more than one splicing event marked by different SACS in the same gene. However, SACS4, SACS5 and SACS7 have a gene overlap of ~40%, which is considerable. Repetition of the GO analysis using unique gene lists at each SACS did not change results dramatically, suggesting that these overlapping genes are not responsible for most of the characteristic biological and KEGG pathway terms found in the analysis (for comparison see Supplementary Figs. 4 and 5).

### Nucleosome positioning

Publicly available MNase based nucleosome positioning at H1 hESC from the NucMap database (http://bigd.big.ac.cn/nucmap/) was used to calculate the nucleosome positioning at SACS-marked exons. SACS exons coordinates were converted from hg19 to hg38 using Liftover from the UCSC Genome Browser Database: Update 2006. Nucleosome positioning average signal was calculated and visualised using Deeptools2^63^, Computematrix and Plotprofile using as reference point the 3’ splice site for each exon.

### Statistical analysis

Statistical analysis and visualisations were performed using R version 3.4.3. and 3.5. In Figs. 4 and 7, we performed Fisher’s exact test and *T*-test, two-sided. In Figs. 5 and 7 and Supplementary Fig. 2, we performed a Wilcoxon Rank test, two tailed, with the following n per each group: in Figs. 5 and 7, H4K20me1 + H3K79me2 exons = 152, H3K14ac + H3K9ac exons=139, H3K9me3 + 5mC Excluded exons = 89, H4K20me1 + H4K91ac exons = 143, H3K9me3 + 5mC, Included exons = 142, H3K4me1 + H3K4me2 exons = 165, non-marked Excluded exons = 600, non-marked Included exons = 600 and Constitutive exons = 600; in Supplementary Fig. 2, Excluded exons *n* = 950, Mid-excluded exons *n* = 332, Mid-included exons *n* = 634 and Included exon *n* = 675. In Fig. 6, the –log10 adjusted *p*-value of the enrichment FDR and associated adjusted *p*-value were calculated from the same number of events as in Figs. 5 and 7. Bonferroni with *n* equal to the number of comparisons was used to correct the obtained *p*-values for multiple pairwise comparisons. All the exact *p*-values can be found at the Source Data file.

### Reporting summary

Further information on research design is available in the Nature Research Reporting Summary linked to this article.

## Supplementary information

Reporting Summary

Supplementary Information

Supplementary Data 1

Supplementary Data 2

Supplementary Data 3

Supplementary Data 4

Supplementary Data 5

Description of Additional Supplementary Files

## Data Availability

All the epigenomics and transcriptomics datasets supporting the findings of this study are available in Supplementary Data 1 and/or within the text/figures. All data is available from the corresponding author upon reasonable request. Source data are provided with this paper.

## Source data

Source Data
