# Supplementary material for: Splicing-associated chromatin signatures: a combinatorial and position-dependent role for histone marks in splicing definition
Source: Nat Commun. 2021 Jan 29;12:682. doi: 10.1038/s41467-021-20979-x (PMC7846797; doi:10.1038/s41467-021-20979-x)
Supplement: Supplementary file 2 — Supplementary Information [file 41467_2021_20979_MOESM2_ESM.pdf]

SPLICING-ASSOCIATED CHROMATIN SIGNATURES: A COMBINATORIAL AND POSITION-DEPENDENT ROLE FOR HISTONE MARKS IN SPLICING DEFINITION

Supplementary Fig. 1. Random Forest (Boruta) results plots in H1 (left) and IMR90 (right) cells.

Supplementary Fig. 2. Enrichment levels of the 15 histone and methyl DNA (5mC) marks selected from the Random Forest classifier.

Supplementary Fig. 3. Specificity and conservation of SACS.

Supplementary Fig. 4. Gene ontology enrichments for the chromatin-marked alternatively spliced genes (SACS).

Supplementary Fig. 5. Gene ontology enrichments for non-overlapping SACS-marked alternatively spliced genes.

SUPPLEMENTARY FIGURE 1

a

Epigenetic features informative to classify included from excluded events in H1

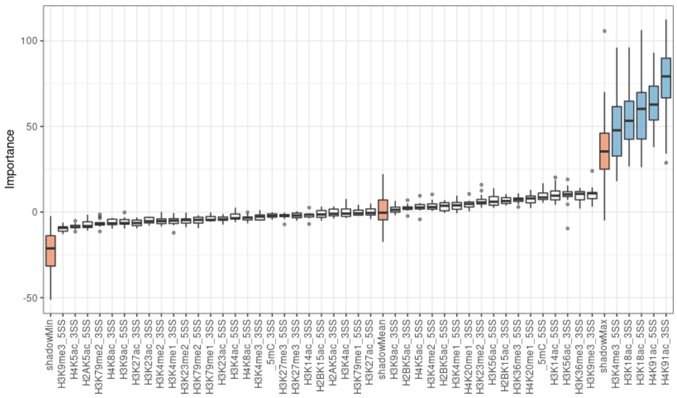

Epigenetic features informative to classify included from excluded events in IMR90

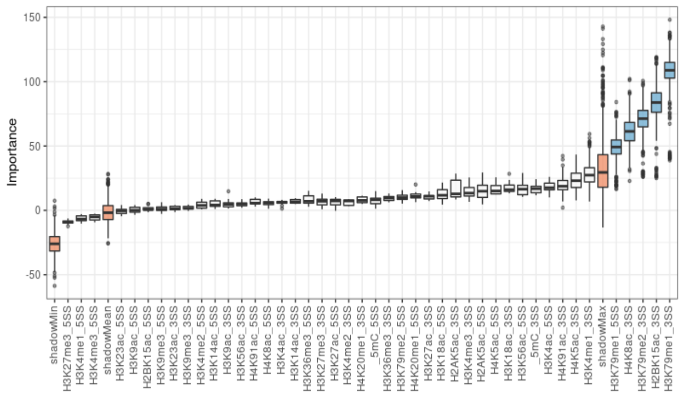

b

Epigenetic features informative to classify included from mid-excluded events in H1

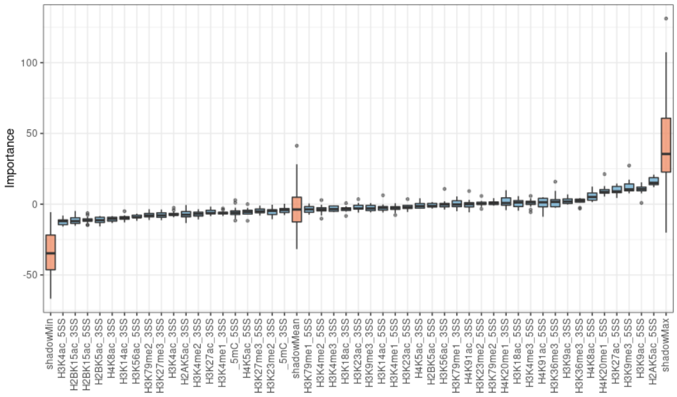

Epigenetic features informative to classify included from mid-excluded events in IMR90

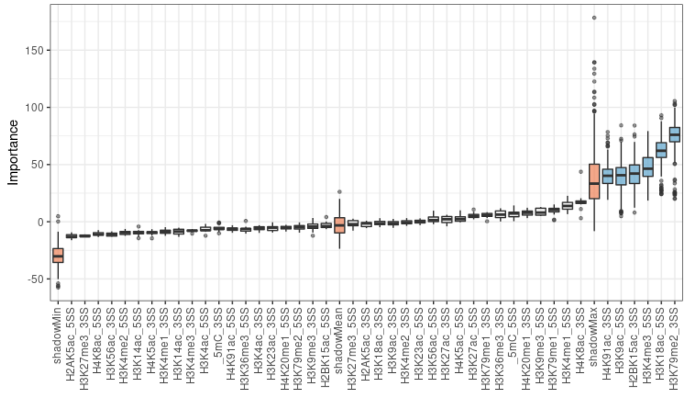

c

Epigenetic features informative to classify included from mid-included events in H1

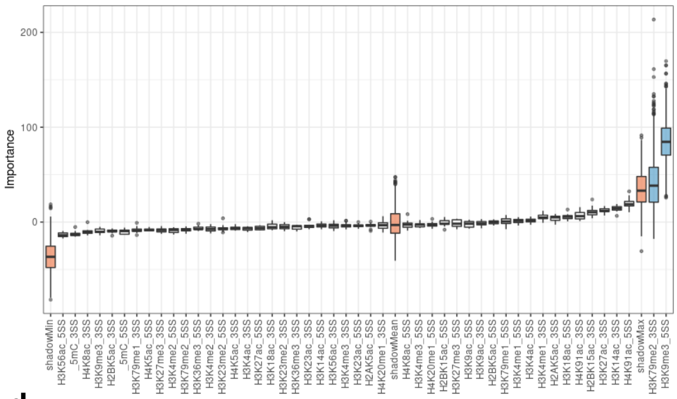

Epigenetic features informative to classify included from mid-included events in IMR90

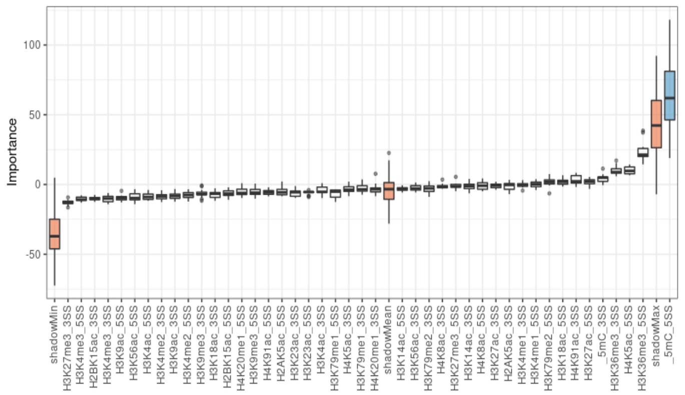

d

Epigenetic features informative to classify excluded from mid-included events in H1

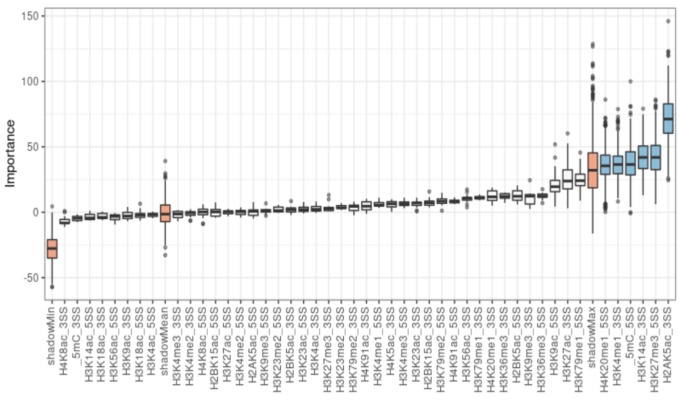

Epigenetic features informative to classify excluded from mid-included events in IMR90

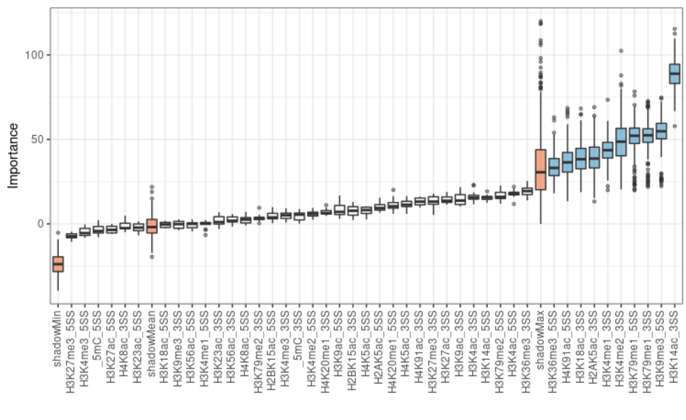

e

Epigenetic features informative to classify excluded from mid-excluded events in H1

Epigenetic features informative to classify excluded from mid-excluded events in IMR90

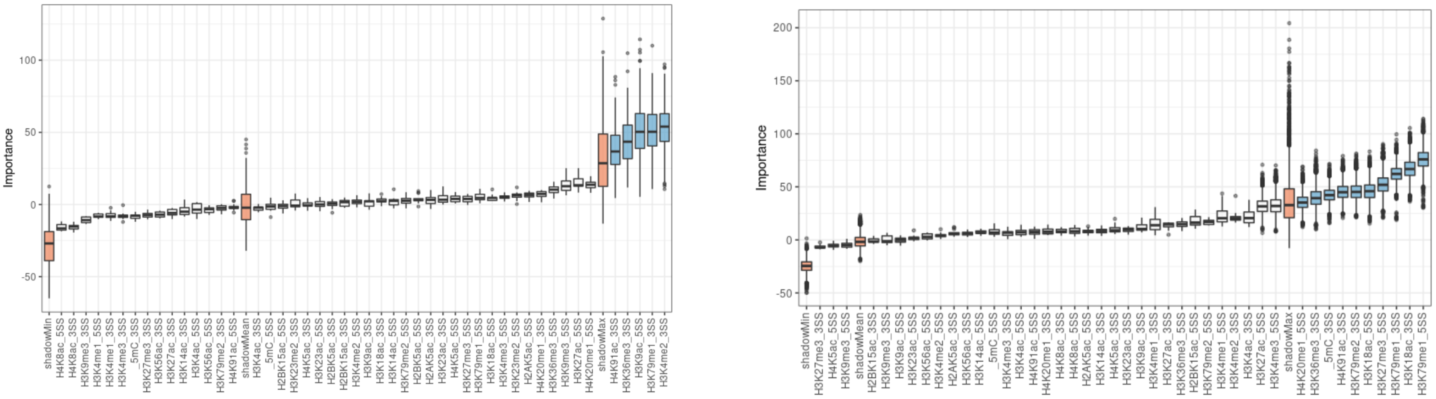

f

Epigenetic features informative to classify mid-excluded from mid-included events in H1

Epigenetic features informative to classify mid-excluded from mid-included events in IMR90

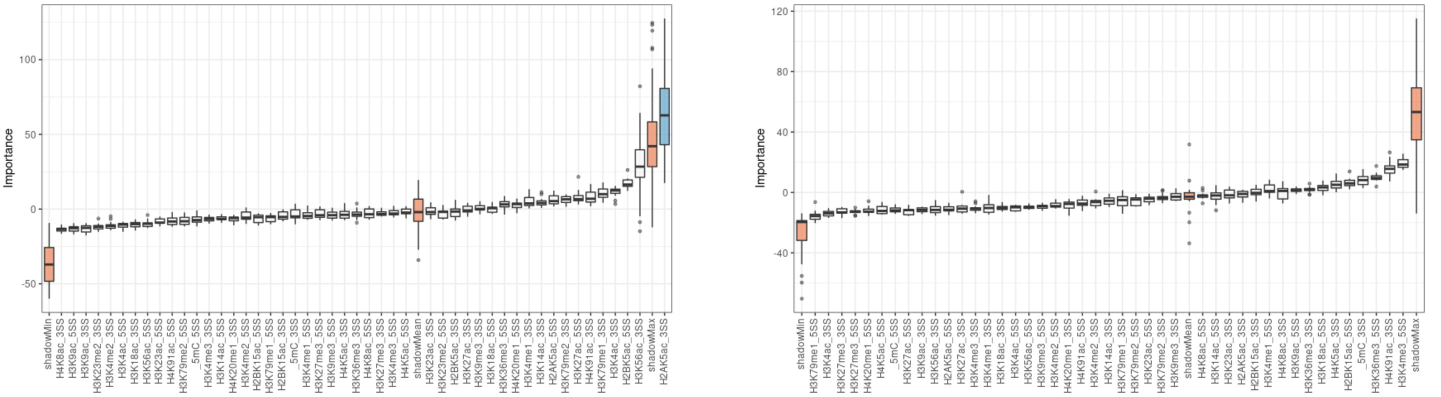

g

Epigenetic features informative to classify randomized splicing events in H1

Epigenetic features informative to classify randomized splicing events in IMR90

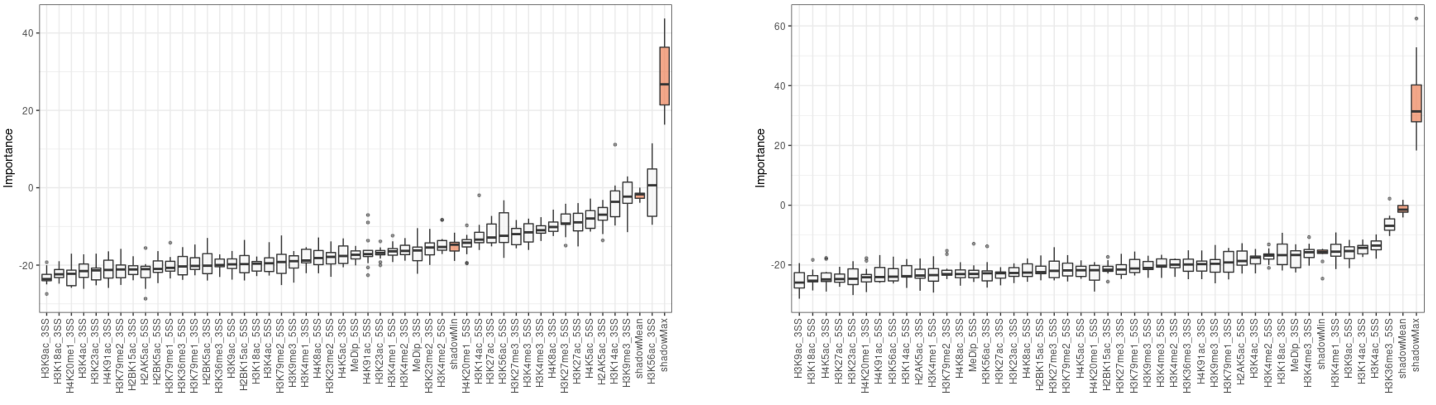

Supplementary Figure 1. Random Forest (Boruta) box plots results in H1 (left) and IMR90 (right) cells for each pair-wise comparison of the four pre-defined splicing groups (included, excluded, mid-included and mid-excluded). The epigenetic features that are informative to classify alternatively spliced exons into one of the four splicing categories are highlighted in blue. In orange are the minimal, average and maximum Importance score of the randomized shadow features used to properly select the informative features. Any feature below the maximum hit of a shadow feature is considered random and discarded. As a control, the same pair-wise analysis was performed with splicing groups in which the inclusion levels were shuffled and randomized (**g**). As expected, no informative feature was obtained. Box plots are centered on the median with interquartile ranges of the importance score.

SUPPLEMENTARY FIGURE 2

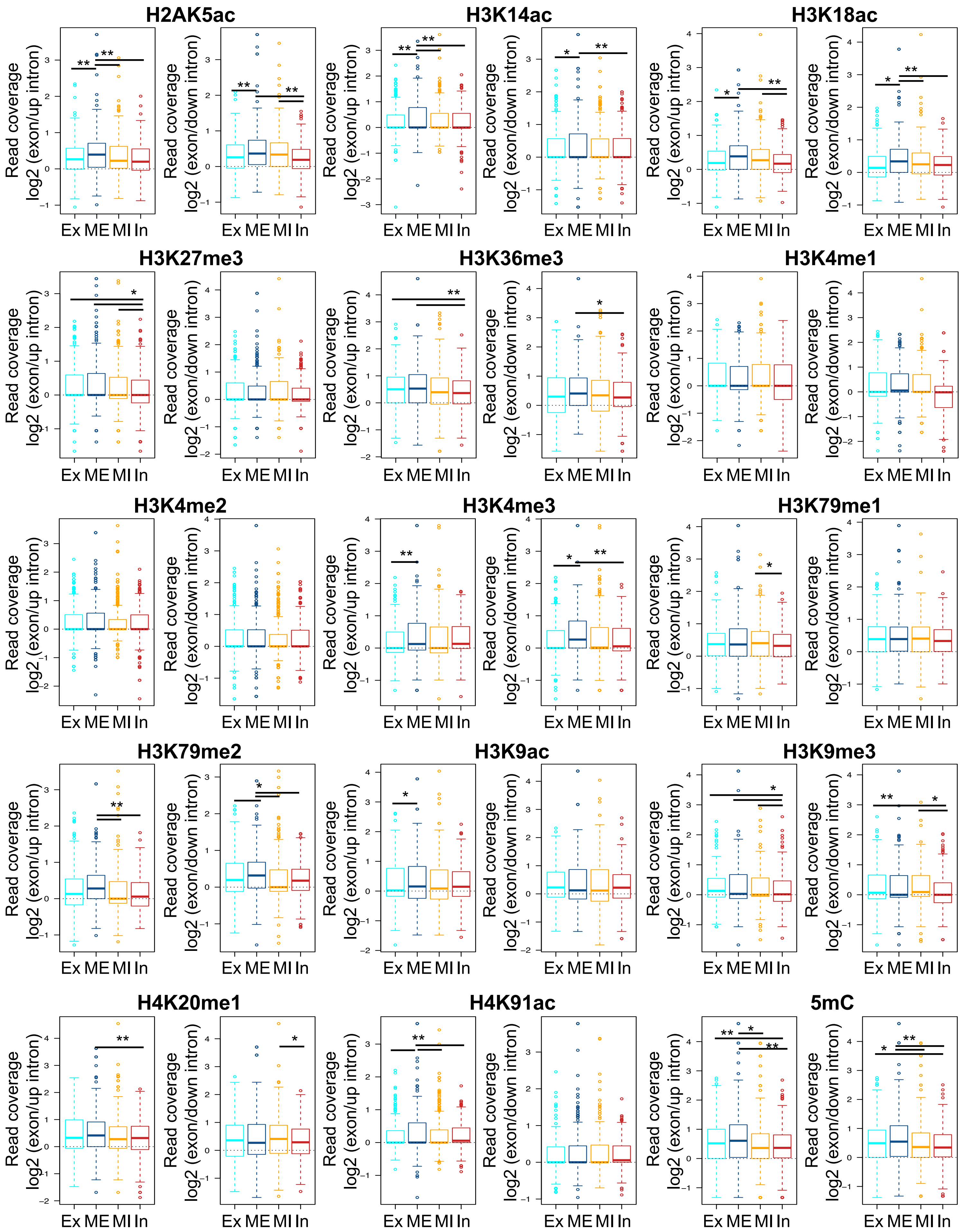

Supplementary Figure 2. Enrichment levels of the 15 histone and methyl DNA (5mC) marks selected from the Random Forest classifier in H1 embryonic stem cells. Box plots centered on the median with interquartile ranges of the log2 (exon/intron) ratio of the normalized read coverage at the exon respect the read coverage upstream (in the left) or downstream (in the right) the intronic region, for each chromatin mark in n=950 excluded (Ex, light blue), n=332 mid-excluded (ME, dark blue), n=634 mid-included (MI, yellow) and n=675 well included (In, red) alternatively spliced events in H1 hESCs. * p-value < 0.05 and ** p-value < 0.01 in Wilcoxon Rank Sum test, two-sided.

SUPPLEMENTARY FIGURE 3

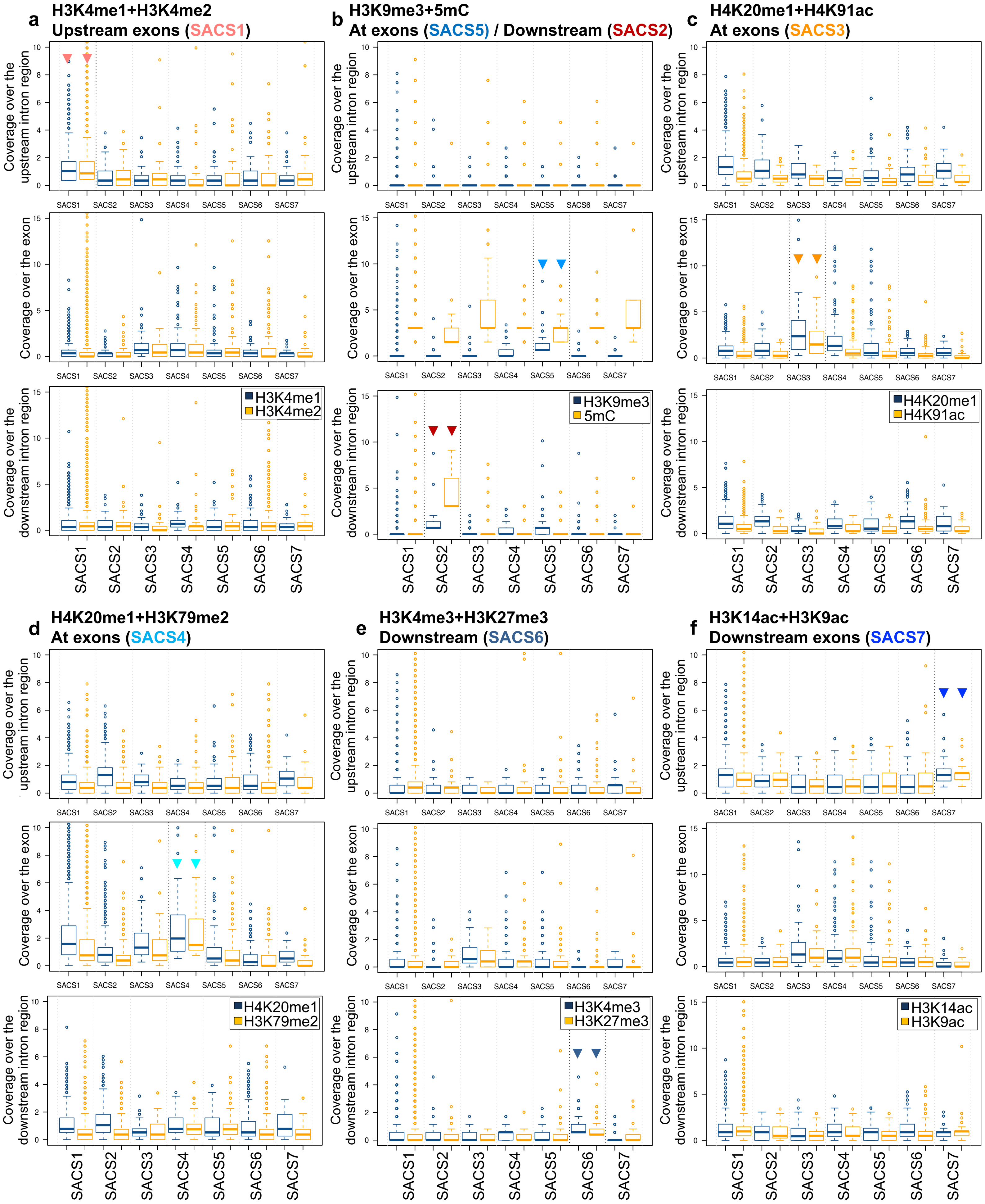

Continue next page

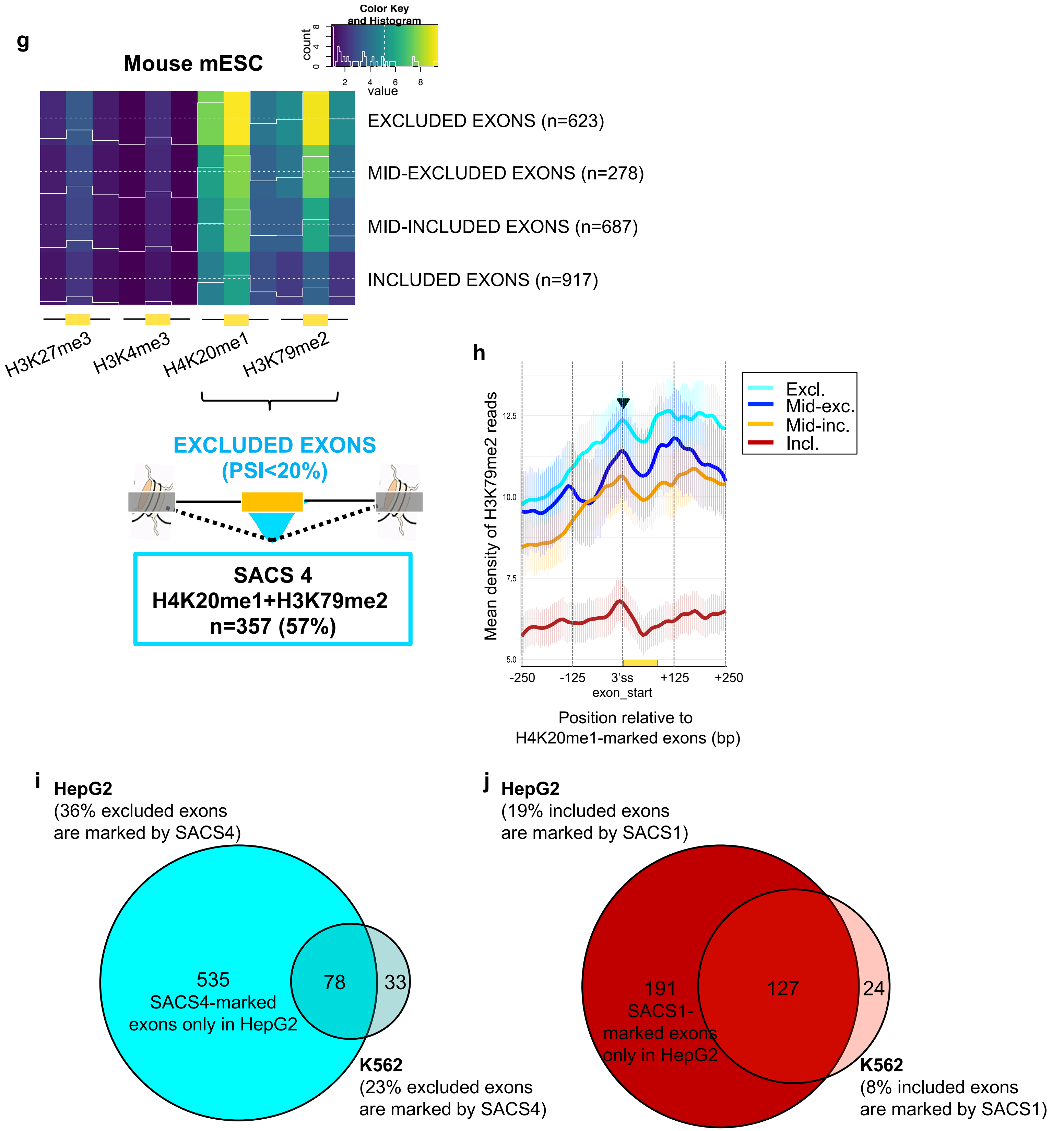

Supplementary Figure 3. Specificity and conservation of SACS. a-f. Box plots centered on the median with interquartile ranges of the normalized read coverage (RPKM) of the designated histone mark 200bp upstream, at the exon and 200bp downstream in SACS-marked exons. A SACS is defined by the two marks co-enriched at a specific position and splicing group. **g-h.** Heatmap of the normalized read coverage upstream, at the exon and downstream the intronic region (represented as a yellow rectangle and a line) for each of the chromatin modifications (H3K27me3, H3K4me3, H4K20me1 and H3K79me2) found in our model and in which there is data available in mouse mESCs. When data from only one of the two chromatin marks from a SACS was available, it was not analysed. Only H4K20me1+H3K79me2 was found to significantly mark exons when excluded, as represented by the SACS4 in human hESCs. Importantly, 57% of all the excluded exons analysed in mouse mESCs where enriched in this signature (357/623). **b.** Density profile of H3K79me2 reads around H4K20me1-marked exons +/- 250 bp from the 3' ss exon start in excluded (Excl.), mid-excluded (Mid-exc.), mid-included (Mid-inc.) and included (Incl.) exons, as represented in Figure 3. **i-j.** Proportional Venn diagrams showing the number of alternatively spliced exons marked by a SACS in HepG2 and K562. In brackets de percentage of chromatin-marked exons respect the total number of exons analyzed per group as in Fig.2a.

SUPPLEMENTARY FIGURE 4

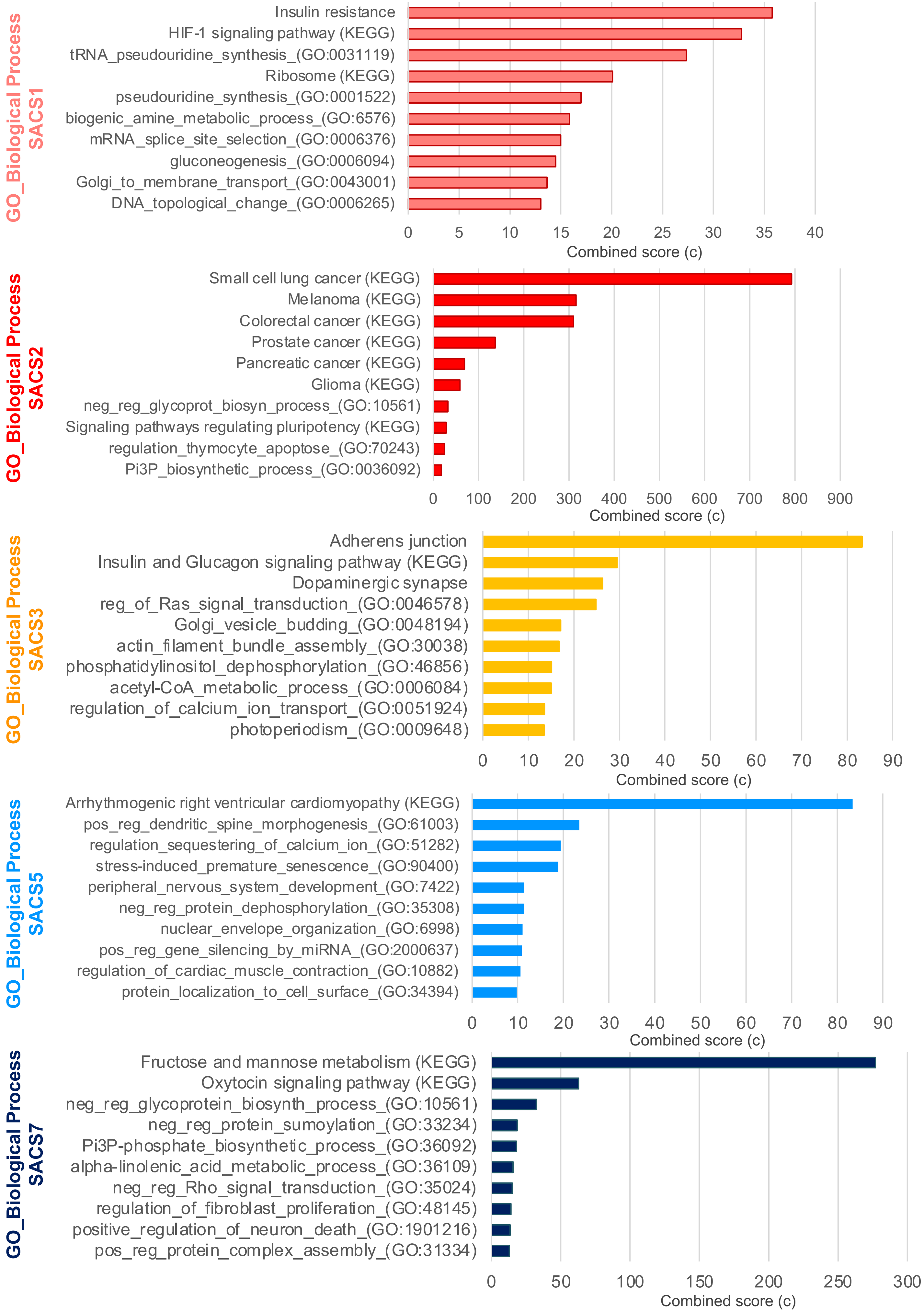

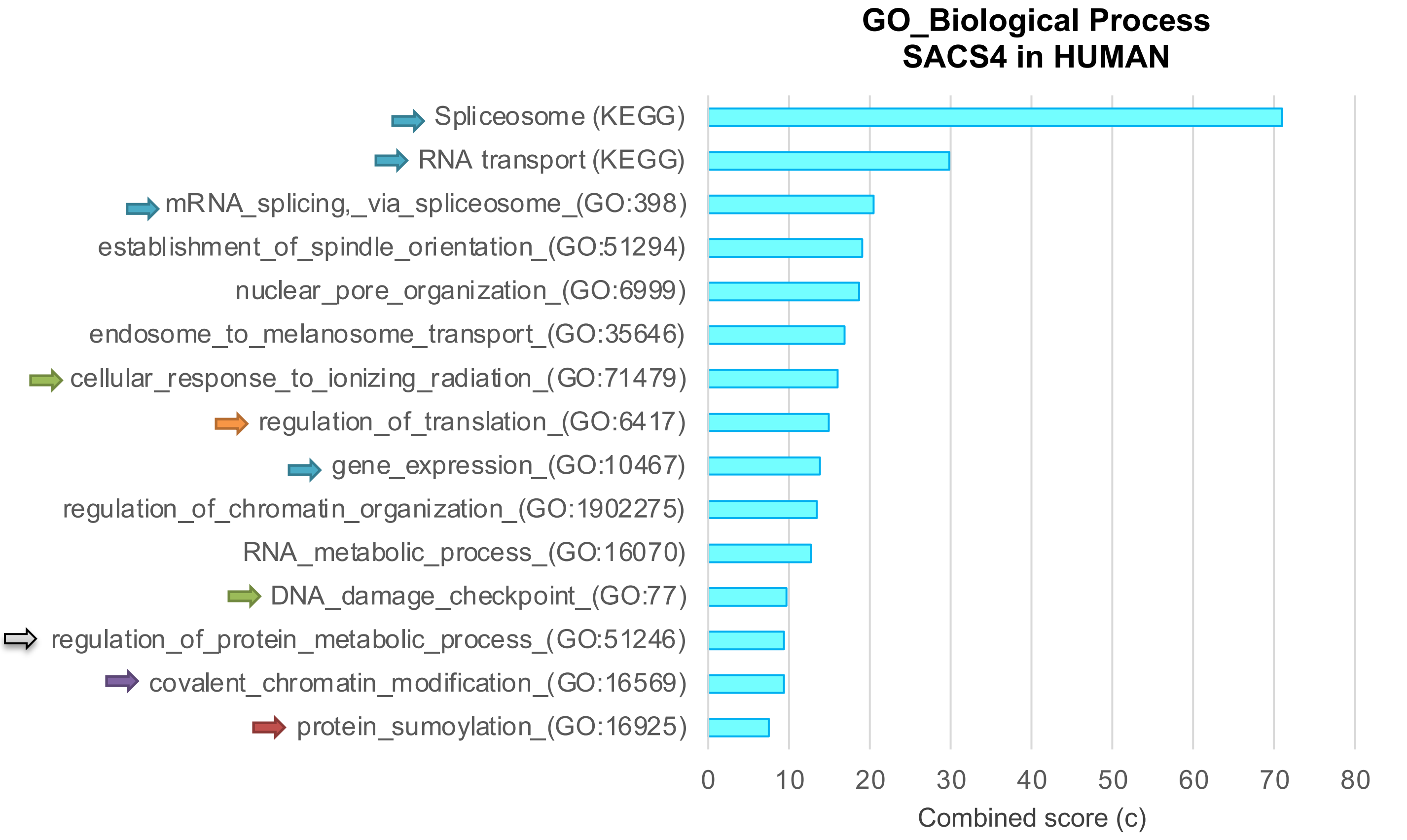

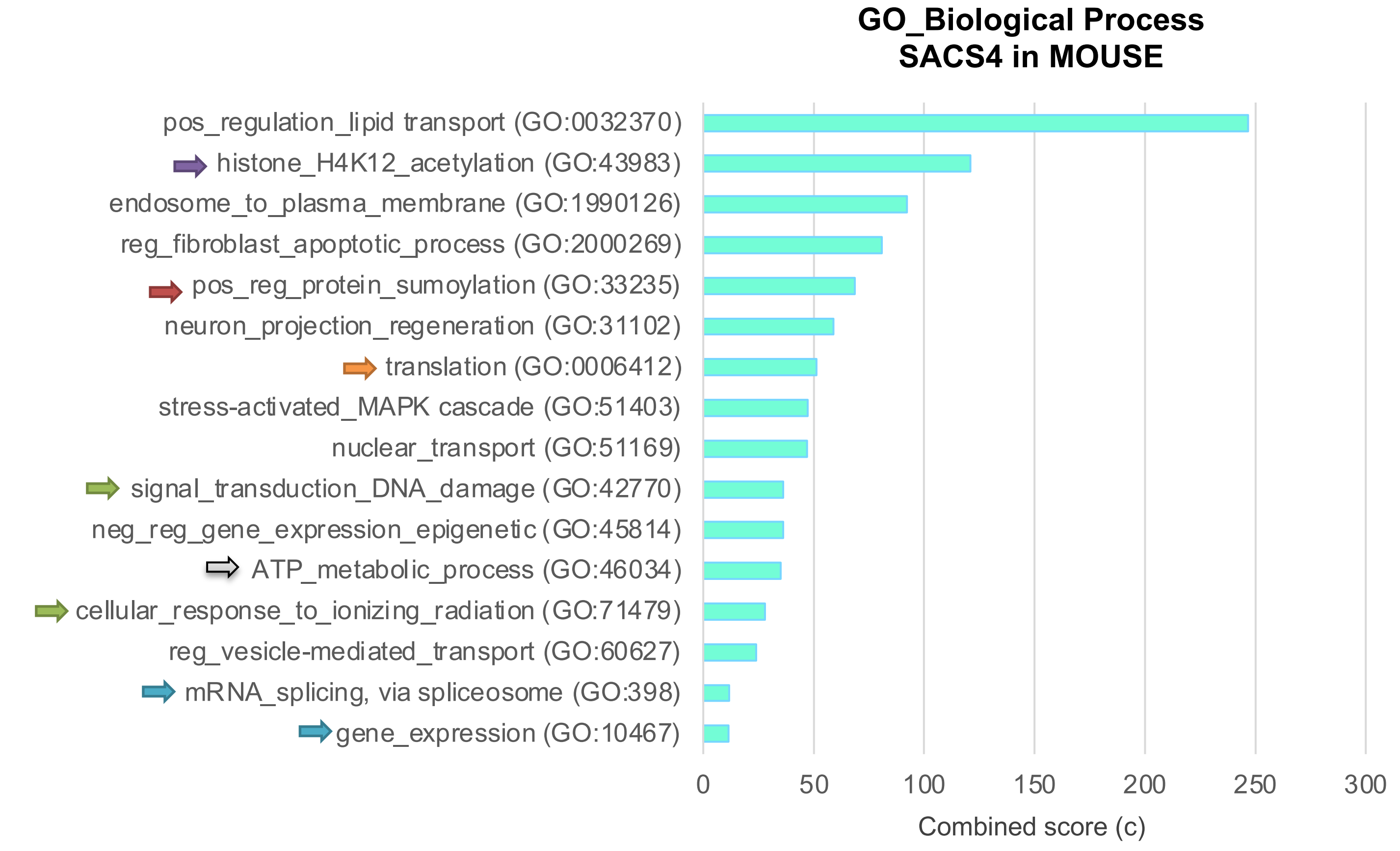

Supplementary Figure 4. Gene ontology (GO) enrichments for the chromatin-marked alternatively spliced genes (SACS). The most significant GO terms related to biological processes are plotted for each SACS using EnrichR combined score as a reference. In SACS4, we highlight with matched colored arrows all the GO terms in common between human hESCs and mouse mESCs. All terms have a p-value < 0.01 in Fisher's exact test, two-sided.

SUPPLEMENTARY FIGURE 5

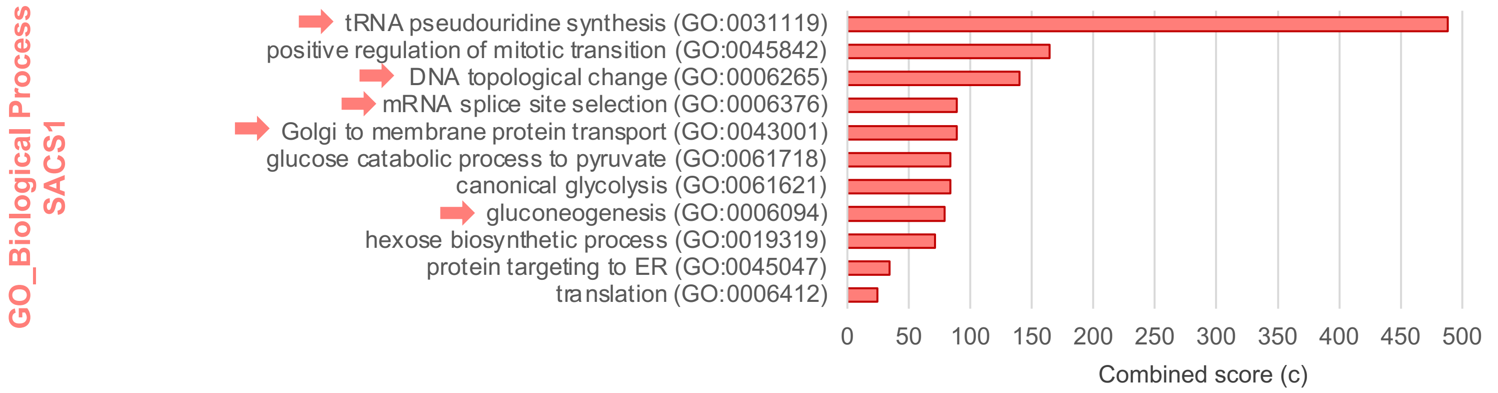

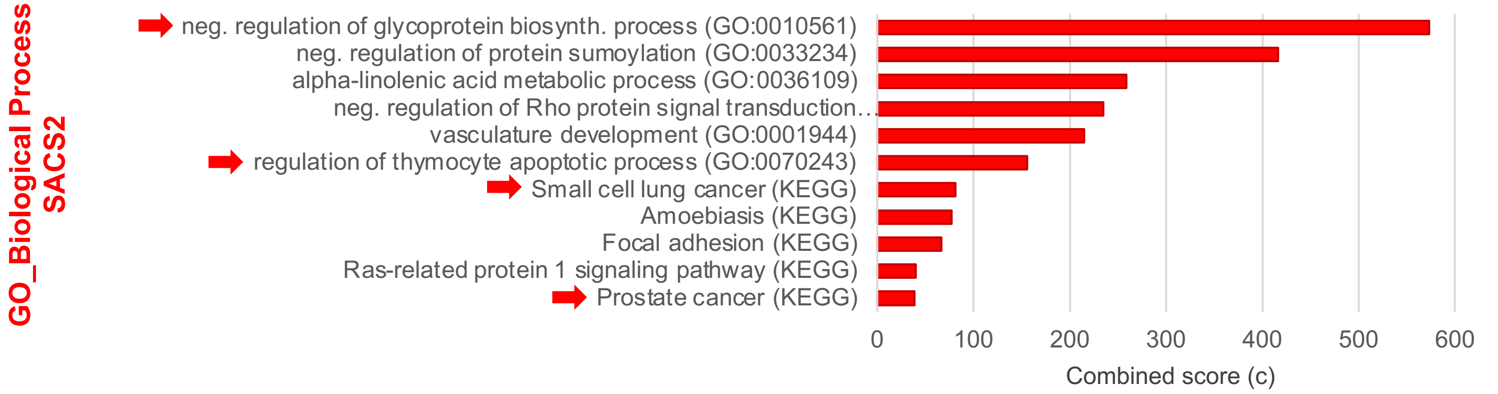

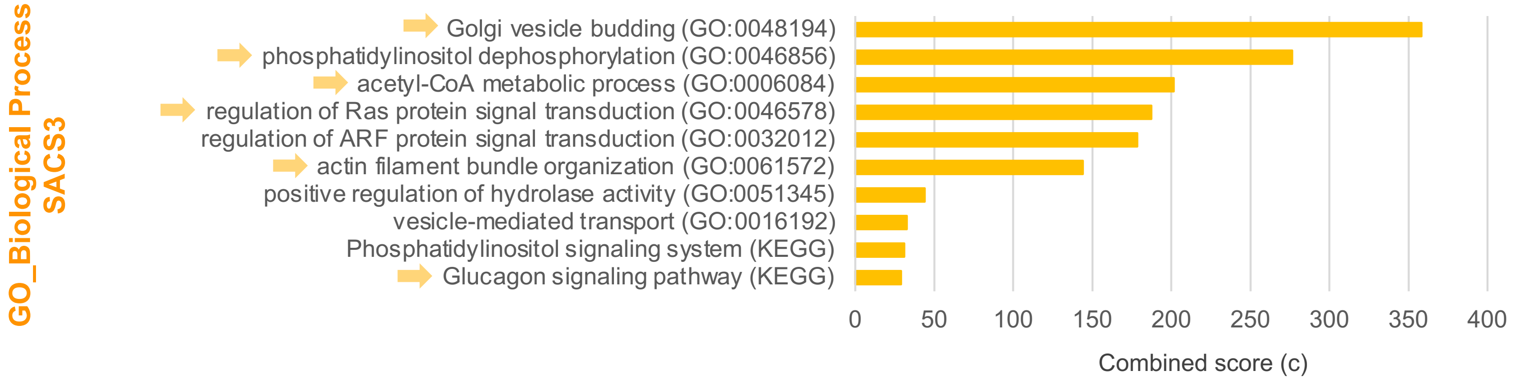

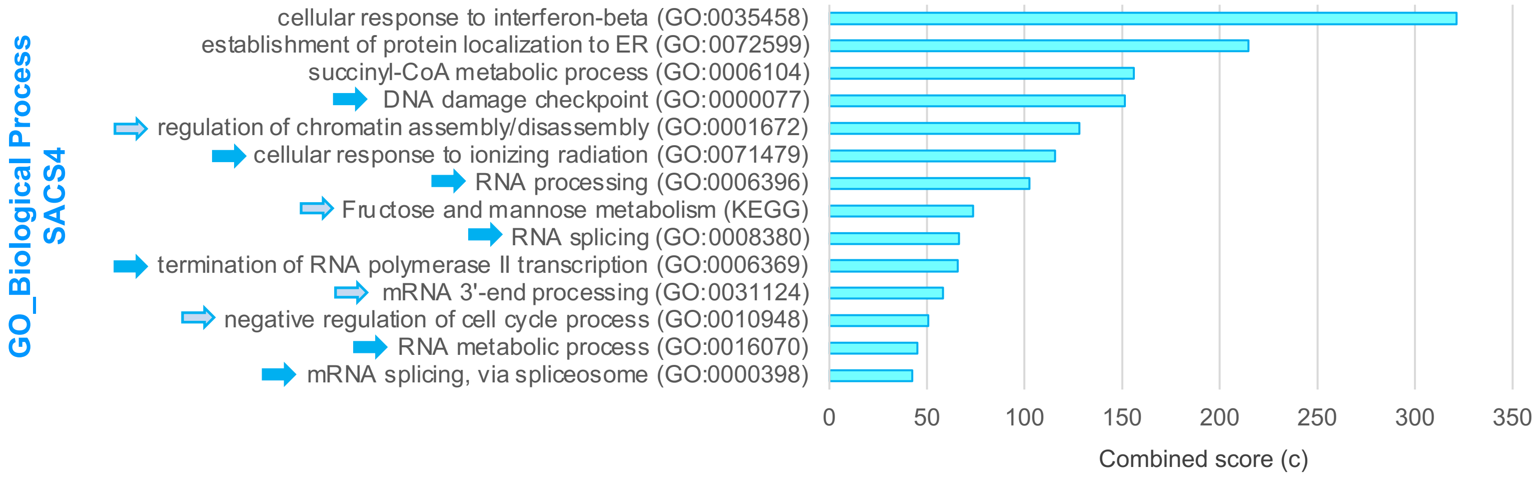

Continue next page

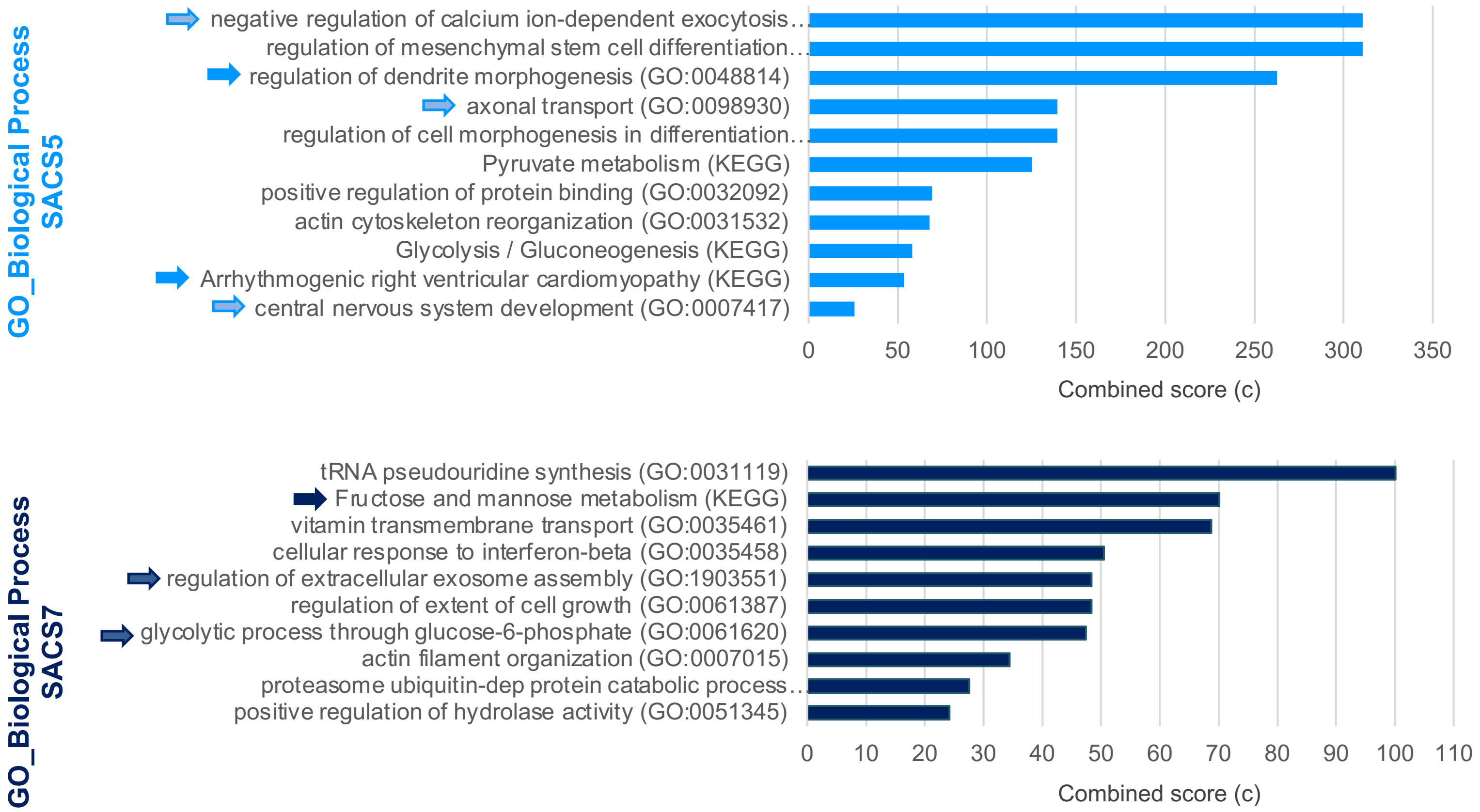

Supplementary Figure 5. Gene ontology (GO) enrichments for non-overlapping SACS-marked alternatively spliced genes. The most significant GO terms related to biological processes are plotted for each SACS with unique non-overlapping genes using EnrichR combined score as a reference. GO terms in common with the previous GO analysis with overlapping genes are highlighted with matched colored arrows. All terms have a p-value < 0.01 in Fisher’s exact test, two-sided.
