# Supplementary material for: Splicing-associated chromatin signatures: a combinatorial and position-dependent role for histone marks in splicing definition
Source: Nat Commun. 2021 Jan 29;12:682. doi: 10.1038/s41467-021-20979-x (PMC7846797; doi:10.1038/s41467-021-20979-x)
Supplement: Supplementary file 8 — Description of Additional Supplementary Files [file 41467_2021_20979_MOESM8_ESM.pdf]

Description of Additional Supplementary Files

Title: Supplementary Data 1.

Description: List of the cell lines and epigenomics and transcriptomics data used from human and mouse. The source and accession number for each data set is given in each category.

Title: Supplementary Data 2.

Description: Random Forest Results. The mean, median, minimum and maximum importance score to classify splicing events into one of the 4 pre-selected splicing groups (Included, Excluded, Mid-Included and Mid-Excluded) in pair-wise comparisons is given for each epigenetic feature in H1 and IMR90 cells. An epigenetic feature is the read density of a chromatin modification at the beginning (3'ss) and end (5'ss) of an alternatively spliced exon. As controls, we used the splice site strength, which is known to classify alternatively spliced exons, and randomized splicing levels. The selected features are ranked for each cell type in two summary sheets.

Title: Supplementary Data 3.

Description: Chromatin pairs defining each SACS. The enrichment scores (based on ChIP-seqs/MeDIP-seqs in H1 hESC) of one mark at regions enriched by the other mark are shown by pairs in both directions (Mark1 and Mark2 Z-scores) for all possible combinations upstream, at the exon and downstream at every splicing group (Excluded, Included, Mid-Included and Mid-Excluded). The 5' and 3' splice sites of each UP and DOWN introns are studied (UP2003SS, UP2005SS, DOWN2003SS, DOWN2005SS). Constitutively spliced exons and exons with randomized splicing levels are used as control (Constitutive and Random). Only the pairs with significant enrichments at the two marks (reciprocal) and in which the enrichment levels at each mark go in the same direction (co-enrichment) are kept. Finally, only the reciprocal co-enriched pairs that are unique for a position and splicing group and that are not found in CONSTITUTIVE or RANDOM exons are kept as a SACS.

Title: Supplementary Data 4.

Description: Lists of alternatively spliced exons marked by a specific splicing-associated chromatin signature (SACS). The list of alternatively spliced exons for each SACS is given together with the list of unique genes for the Gene Ontology analysis. We provide for each SACSmarked exon (in order of appearance): the chromosome, the coordinates in hg19 of the flanking introns, the strand specificity and the gene name.

Title: Supplementary Data 5.

Description: List of the primer pairs used in RT-qPCR and ChIP-qPCR. The name of the gene and primer sequences are detailed
